# Demographic History and Genetic Adaptation in the Himalayan Region Inferred from Genome-Wide SNP Genotypes of 49 Populations

**DOI:** 10.1093/molbev/msy094

**Published:** 2018-05-22

**Authors:** Elena Arciero, Thirsa Kraaijenbrink, Asan, Marc Haber, Massimo Mezzavilla, Qasim Ayub, Wei Wang, Zhaxi Pingcuo, Huanming Yang, Jian Wang, Mark A Jobling, George van Driem, Yali Xue, Peter de Knijff, Chris Tyler-Smith

**Affiliations:** 1 The Wellcome Sanger Institute, Wellcome Genome Campus, Hinxton, United Kingdom; 2 Department of Human Genetics, Leiden University Medical Center, Leiden, The Netherlands; 3 BGI-Shenzhen, Shenzhen, China; 4 Division of Experimental Genetics, Sidra Medical and Research Center, Doha, Qatar; 5 Tropical Medicine and Biology Multidisciplinary Platform, Monash University Malaysia Genomics Facility, Selangor Darul Ehsan, Malaysia; 6 School of Science, Monash University Malaysia, Selangor Darul Ehsan, Malaysia; 7 The Third People’s Hospital of the Tibet Autonomous Region, Lhasa, China; 8 James D. Watson Institute of Genome Science, Hangzhou, China; 9 Department of Genetics & Genome Biology, University of Leicester, Leicester, United Kingdom; 10 Institute of Linguistics, University of Bern, Bern, Switzerland

**Keywords:** Himalayas, human population history, high-altitude adaptation, positive selection, Indo-European language, Tibeto-Burman language

## Abstract

We genotyped 738 individuals belonging to 49 populations from Nepal, Bhutan, North India, or Tibet at over 500,000 SNPs, and analyzed the genotypes in the context of available worldwide population data in order to investigate the demographic history of the region and the genetic adaptations to the harsh environment. The Himalayan populations resembled other South and East Asians, but in addition displayed their own specific ancestral component and showed strong population structure and genetic drift. We also found evidence for multiple admixture events involving Himalayan populations and South/East Asians between 200 and 2,000 years ago. In comparisons with available ancient genomes, the Himalayans, like other East and South Asian populations, showed similar genetic affinity to Eurasian hunter-gatherers (a 24,000-year-old Upper Palaeolithic Siberian), and the related Bronze Age Yamnaya. The high-altitude Himalayan populations all shared a specific ancestral component, suggesting that genetic adaptation to life at high altitude originated only once in this region and subsequently spread. Combining four approaches to identifying specific positively selected loci, we confirmed that the strongest signals of high-altitude adaptation were located near the Endothelial PAS domain-containing protein 1 and Egl-9 Family Hypoxia Inducible Factor 1 loci, and discovered eight additional robust signals of high-altitude adaptation, five of which have strong biological functional links to such adaptation. In conclusion, the demographic history of Himalayan populations is complex, with strong local differentiation, reflecting both genetic and cultural factors; these populations also display evidence of multiple genetic adaptations to high-altitude environments.

## Introduction

The Greater Himalayan Region is a geographical area containing the world’s highest mountain peaks and a diversity of environments that have required substantial genetic adaptations by the humans who live there. This mountain barrier has also shaped the genetic, cultural, and ethnolinguistic mosaic of South and East Asia. At present, the area falls into the countries of Nepal, Bhutan, India, Pakistan, and the Tibetan Plateau in China. Opinions are divided about whether the Himalayas were used as a corridor that facilitated human migrations from the Tibetan plateau to South Asia in ancient times, or alternatively remained uninhabited due to their inhospitality until more recent times (Majumder 2008; Gayden et al. 2009, 2013; Qi et al. 2013). Archaeological data suggest that the central Tibetan Plateau was populated during the Neolithic period (Meyer et al. 2017), and there is evidence of earlier human occupation in the north-eastern Qinghai region (Aldenderfer 2011).

The Himalayan region is also one of the most complex linguistic areas in the world, containing six linguistic phyla with multiple languages within each phylum, and at least two language isolates (Burushaski and Kusunda) (van Driem 2001; Kraaijenbrink et al. 2014). However, the region has not been fully represented in genetic studies overall. Previous analyses have mainly focused on populations residing to the north or south of this area, or on small numbers of populations (Gayden et al. 2009; Cai et al. 2011; Kang et al. 2012; Jeong et al. 2014; Cole et al. 2017). In the first systematic survey of Himalayan populations, which used autosomal microsatellite markers (STRs) (Kraaijenbrink et al. 2014), we showed higher genetic diversification among the Himalayans compared with the populations from the surrounding regions, and observed genetic differentiation between Indo-European and Tibeto-Burman speakers, suggesting that both language and geography have influenced the genetic structure of these populations. Genomic scans in Tibetans, and Sherpa from Nepal, have previously identified genomic regions associated with high-altitude adaptation. In particular, a derived Endothelial PAS domain-containing protein 1 (*EPAS1*) haplotype, whose frequency is strongly correlated with altitude in the Himalayan populations, has been suggested to have been acquired from an extinct hominin species, Denisovans (Yi et al. 2010; Huerta-Sanchez et al. 2014; Lorenzo et al. 2014; Hackinger et al. 2016). In the current study, we have performed a genome-wide SNP-based analysis of 738 individuals from 49 populations in the region in order to generate a more comprehensive reference data set, further understand the population structure and demographic history of the area, as well as search more widely for positively selected genomic regions.

## Results

### Himalayan Samples Show Distinct Patterns of Population Structure

**Fig. 1.**
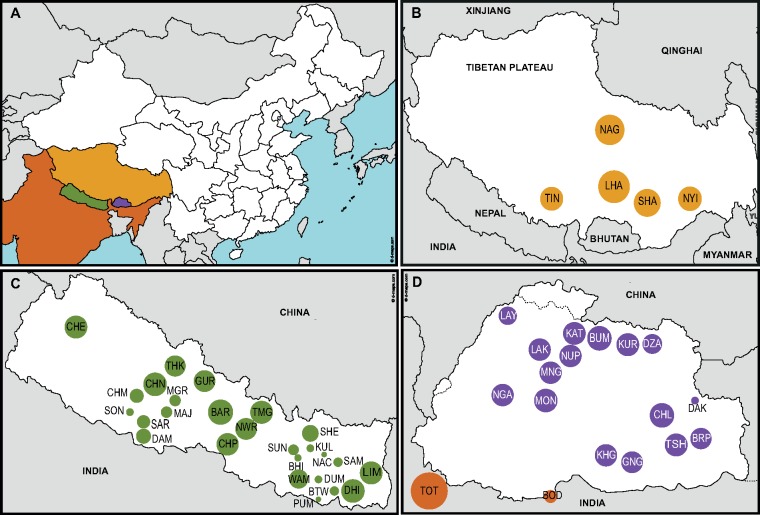
Population samples analyzed in this study. (*A*) Map of South and East Asia, highlighting the four regions examined, and the colour assigned to each. (*B*) Samples from the Tibetan Plateau. (*C*) Samples from Nepal. (*D*) Samples from Bhutan and India. The circle areas are proportional to the sample sizes. The three letter population codes in (*B–D*) are defined in supplementary table S1, Supplementary Material online.

**Fig. 2.**
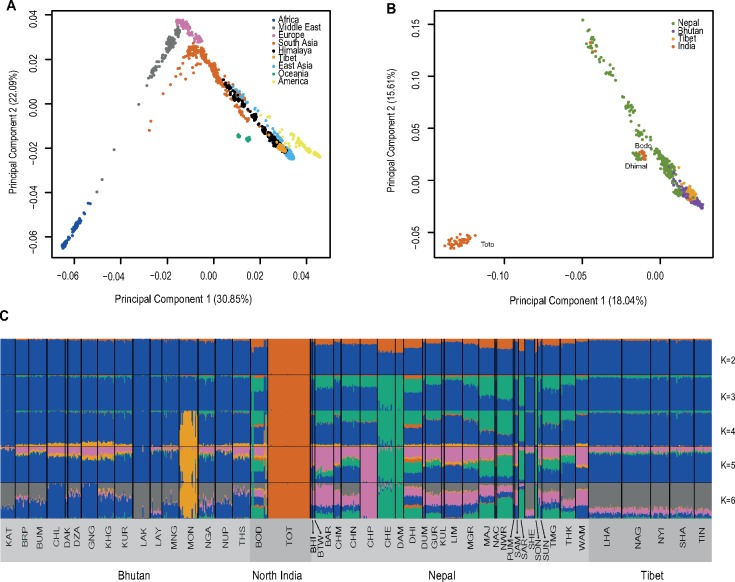
Genetic structure of the Himalayan region populations from analyses using unlinked SNPs. (*A*) PCA of the Himalayan and HGDP-CEPH populations. Each dot represents a sample, coded by region as indicated. The Himalayan region samples lie between the HGDP-CEPH East Asian and South Asian samples on the right-hand side of the plot. (*B*) PCA of the Himalayan populations alone. Each dot represents a sample, coded by country or region as indicated. Most samples lie on an arc between Bhutanese and Nepalese samples; Toto (India) are seen as extreme outlier in the bottom left corner, while Dhimal (Nepal) and Bodo (India) also form outliers. (*C*) ADMIXTURE (*K* values of 2–6, as indicated) analysis of the Himalayan samples. Note that most increases in the value of *K* result in single population being distinguished. Population codes in (*C*) are defined in supplementary table S1, Supplementary Material online.

We first investigated the population history and demography of the region (fig. 1 and supplementary table S1, Supplementary Material online) by determining the genetic relationships among the Himalayan populations, and comparing them with published data sets of 78 worldwide populations (supplementary table S1, Supplementary Material online). Principal Components Analysis (PCA) shows that the Himalayan populations form a cline, lying between the South and East Asian samples. Populations from Nepal are close to Indians, whereas those from Bhutan and Tibet are closer to East Asians (fig. 2*A* and supplementary fig. S1, Supplementary Material online). This pattern of genetic affinity to South and East Asian populations is also supported by an ADMIXTURE analysis of worldwide populations (supplementary fig. S2, Supplementary Material online), where the genetic component from South Asia (orange) is observed particularly in the Nepalese, and the East Asian (gold) component in the Nepalese, as well as the Bhutanese and Tibetans. However, except for the Toto, all other Himalayan populations are mainly characterized by their own ancestral component (blue). We also found some detectable European and Middle Eastern ancestral components (off-white and green) in some Nepalese. On a finer scale, the first component of a PCA using only the Himalayan populations shows strong geographical clustering with the Toto population forming an outlier, while the second principal component identifies substructure within the Himalayan populations (fig. 2*B*). Individuals from Nepal lie in several dispersed clusters, whereas those from Bhutan and Tibet group together. Interestingly, the Nepalese Sherpa cluster with the Tibetans and some Bhutanese populations from high altitude. A distinct cluster is formed by Dhimal and Bodo individuals from Nepal and North India, respectively (fig. 2*B* and supplementary fig. S3, Supplementary Material online). The ADMIXTURE analysis using only the Himalayan populations shows patterns consistent with the PCA, with different proportions of ancestral components between Nepal, Bhutan, North India, and Tibet (fig. 2*C*). Each increase in the value of *K* between 2 and 5 usually leads to a single population being distinguished, suggesting extensive genetic isolation and drift. Toto, an outlier in the PCA, is also characterized by an independent ancestral component even at a *K* value of 2 (fig. 2*C*). By contrast, the five Tibetan populations do not show any substructure in this analysis. The lowest CV error was at a *K* value of 6, where we observe a single widespread ancestral component (gray) which is shared among all the high-altitude populations and is significantly positively correlated with altitude (rho = 0.79; *P* = 2.2×10^−18^) (supplementary fig. S4, Supplementary Material online).

A long-term Ne value can be estimated using SNP genotyping data, but has limitations and can only be used as a proxy for the variability of their effective population sizes and thus the overall genetic diversity, but nevertheless allows some informative comparisons. The Chetri have the highest long-term Ne, whereas Toto have the lowest (supplementary fig. S5, Supplementary Material online), suggesting that the low genetic variation in Toto could be due to genetic drift or endogamy (Newman and Pilson 1997). All Tibetan populations display similar population sizes (supplementary fig. S5, Supplementary Material online) (de Roos et al. 2008). The identification of population split times shared the same limitations as the Ne estimates, but the sequence of splits suggests that the Himalayans separated first from Indian populations (with possible exceptions of Chetri, Damai, and Sarki), then from East Asians and finally among themselves. Interestingly, all of the high-altitude populations in this data set display a similar differentiation time from other Himalayans, and place this at ∼6,000–5,000 years ago (supplementary fig. S6 and table S2, Supplementary Material online). Despite the limitation of the approach we used, this estimate is in line with several previous genetic and linguistic estimates (Wang 1998; Hu et al. 2017; Zhang et al. 2017), but differs from others (Yi et al. 2010; Aldenderfer 2011; Qi et al. 2013; Lu et al. 2016). The various Tibetan populations display very recent split times from each other, which is consistent with the lack of substructure within these populations.

We explored whether or not Himalayan populations show extended runs of homozygosity (ROHs), which may arise from endogamy. Overall, Himalayan populations are characterized by a high number of autozygous segments of different lengths across the genome (Lu et al. 2016). Nepalese and Bhutanese populations show the most numerous ROHs, and these are also the longest, up to ∼80 and ∼90 Mb in length, respectively. Toto from India are characterized by the highest number of individual ROHs up to ∼50 Mb in length. On the other hand, Tibetan populations show the lowest number and length of ROHs (supplementary fig. S7 and table S2, Supplementary Material online). The total length of ROHs per sample correlates positively with the coefficient of inbreeding (F) (supplementary fig. S8, Supplementary Material online). Bhutanese, Indian, and Nepalese populations show the highest coefficient of inbreeding values and have total lengths of ROHs ∼300–400 Mb. Tibetans show a very low coefficient of inbreeding associated with low numbers of ROHs. Overall, the number and length of ROHs in Himalayan populations are in line with those in other worldwide populations: in such a comparison, Toto show the highest numbers, followed by American and Middle Eastern populations, while Bhutanese populations show a total length and number of ROHs similar to populations from South Asia (supplementary fig. S9 and table S2, Supplementary Material online).

**Fig. 3.**
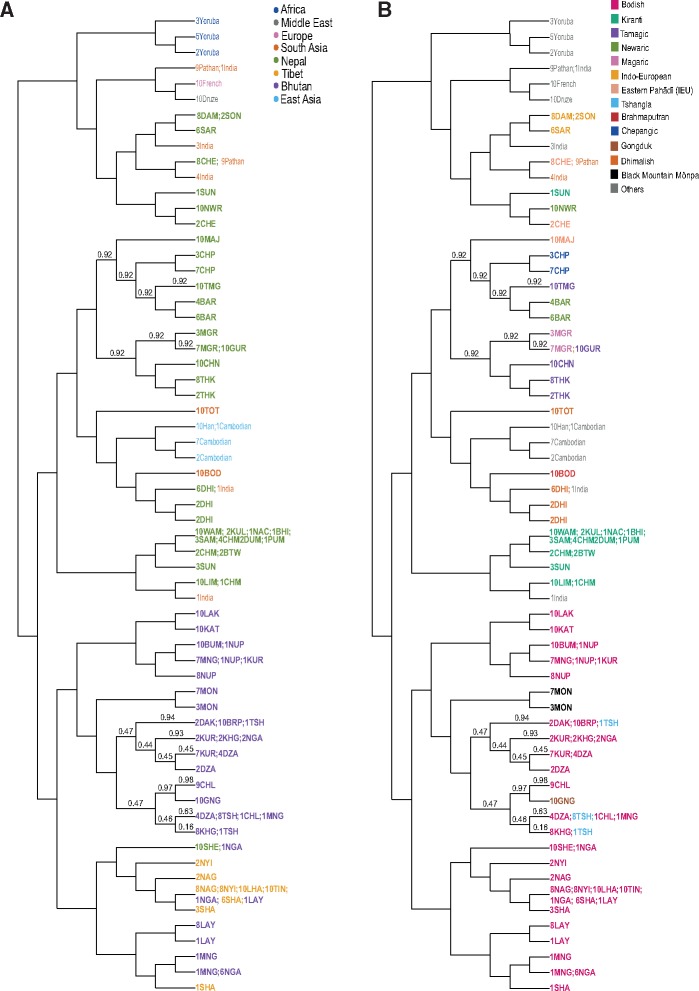
Genetic structure of the Himalayan populations from haplotype analysis using fineSTRUCTURE, and comparison with language. (*A*) Populations are clustered according to haplotype sharing; the branching pattern represents this hierarchy, but the branch lengths have no meaning. Note the geographical clustering of populations, particularly the Bhutanese. (*B*) Language family annotation of the genetic clusters revealing correspondences between genetics and language. Population codes are defined in supplementary table S1, Supplementary Material online.

The phased Himalayan and worldwide population data were also used to reconstruct phylogenetic relationships between the samples and to identify population structure through a Bayesian clustering algorithm implemented in fineSTRUCTURE. The inferred phylogenetic tree shows two main branches splitting Nepalese from Bhutanese plus Tibetans (fig. 3*A*). All the Himalayan high-altitude populations, including the Tibetans, cluster together, with the exception of the Thakali population from Nepal, which clusters with its Nepalese neighbours. Within genetic clusters of the Nepalese and Bhutanese it is possible to recognize substructure based on population and linguistic features. This tree topology was replicated when fineSTRUCTURE was applied to a data set comprising only Himalayan and 1000 Genomes Project Phase 3 populations, which allowed a higher number of SNPs to be used (supplementary fig. S10, Supplementary Material online). PCA was also calculated from the coancestry matrix generated by fineSTRUCTURE confirming that the Himalayan populations are distributed along a cline with the Sherpa, Bhutanese, and Tibetans clustering together (supplementary fig. S11, Supplementary Material online). Comparing the genetic tree with the linguistic affiliation of each Himalayan population (fig. 3*B*), we see that in particular in Bhutan there is agreement between genetic and linguistic subdivisions. Speakers of Kiranti languages from Nepal form a separate cluster, and their languages constitute a distinct linguistic subgroup within the Tibeto-Burman language family. Dhimal from Nepal and Bodo from North India form a separate branch, supporting the PCA result, but not the traditionally accepted language affiliation, and also correspond well with a new linguistic hypothesis which groups Dhimal and the Bodo-Koch languages together within a “Brahmaputran” subgroup (van Driem 2001).

Finally, we computed *D*-statistics (Yoruba, Han; high-altitude Himalayan 1, high-altitude Himalayan 2) for pairs of Sherpa, Tibetan, and Bhutanese populations (Jeong et al. 2017). *D*-statistics values were close to zero for most of the pairs (0.0001≤|*D-*statistic|≤0.0061), with just 36 out of 210 tests statistically significant at a Z score ≥4 (values 0.072≤|Z|≤7.656), showing that some high-altitude Himalayan populations have increased genetic affinity with the low-altitude East Asians (supplementary table S3 and fig. S12, Supplementary Material online). However, unlike the Tibetan samples in Jeong et al. (2017), our Himalayan populations do not follow a longitudinal cline (or a latitudinal one) related to their genetic affinity to low-altitude East Asians (Mantel test *r* = 0.15 and *P* value = 0.18 for longitude, *r* = 0.11 and *P* value = 0.18 for correlation with latitude). This difference may reflect the smaller range of longitude of our samples.

### Complex Demographic History in the Himalayas

**Fig. 4.**
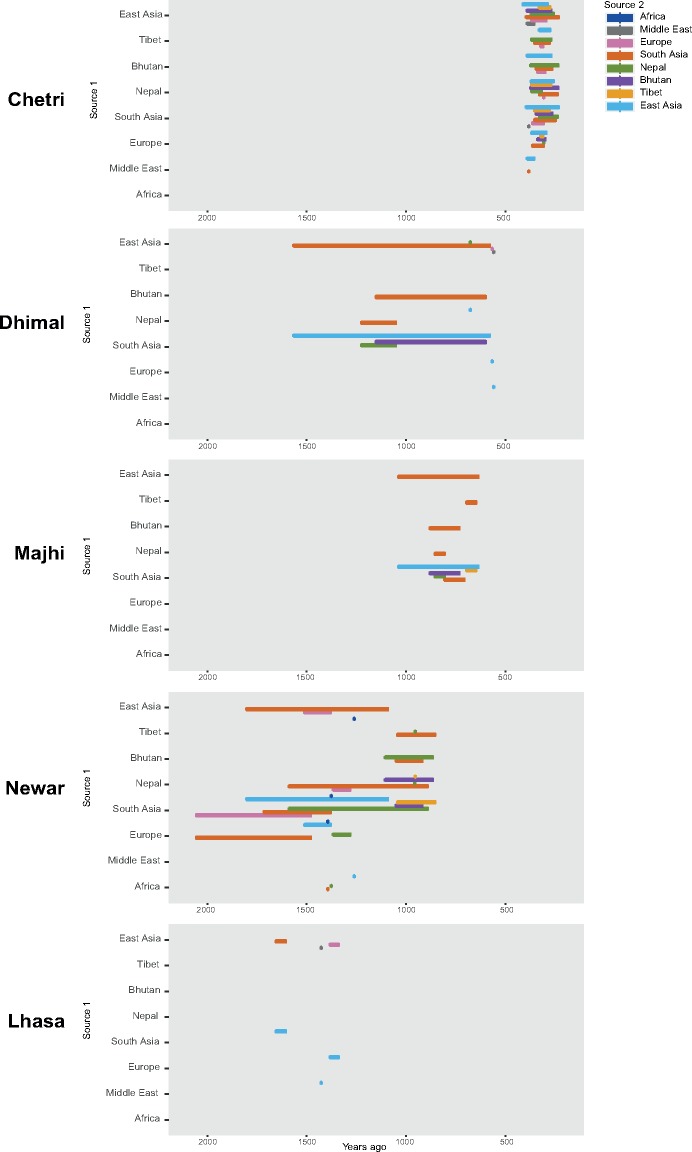
Admixture history of five Himalayan populations. The five populations, each named on the left, could be modelled as a mixture between different source populations from two regions. One of these is shown on the vertical axis, while the second is indicated by the colour of the horizontal bar; the position of this bar represents the inferred time of admixture, and the length in time of these admixture events, according to the scale on the horizontal axis. Thus, the Chetri, for example, can be modelled as a mixture of a large number of Asian and European pairs of populations, occurring ∼200–400 years ago.

We studied gene flow and admixture between Himalayan and nearby populations through three approaches: *f3*-statistics, ALDER, and TreeMix. All the tests provide evidence of admixture between Himalayan and other populations (fig. 4 and supplementary fig. S14 and table S3, Supplementary Material online). Overall, Himalayan populations are characterized by gene flow within the region and with neighbouring populations from South and East Asia. The *f3*-statistics and ALDER show significant admixture events with the Nepalese, North Indians, and Tibetans from China, South Asia, the Middle East, and Europe (fig. 4 and supplementary table S3, Supplementary Material online). ALDER also detected extra, although limited, admixture events between the Bhutanese and populations from South and East Asia ∼800 and 900 years ago (supplementary table S3, Supplementary Material online). Furthermore, Chetri, Majhi, Newar, Dhimal, Bodo, and Lhasa show gene flow from Europe and the Middle East that might be attributed to the presence of these western components as part of the Ancestral North Indian component in South Asians (Reich et al. 2009; Metspalu et al. 2011; Moorjani et al. 2013). Chetri, Bodo, Majhi, and Dhimal show a signature of admixture dated to between 1,000 and 200 years ago. Newar and Lhasa display older signatures of gene flow dated between 1,000 and 2,000 years ago (fig. 4 and supplementary table S3, Supplementary Material online). TreeMix analysis shows long branches for the Toto, Mönpa, and Chepang populations in agreement with the genetic drift patterns (supplementary fig. S14, Supplementary Material online). This is supported by the lack of detectable admixture events for these populations with *f3*-statistics and only a few significant results for Toto with ALDER, showing an admixture event ∼600–800 years ago with Chinese and Indian populations (supplementary table S3, Supplementary Material online). Migration edges involving populations from South and East Asia are detectable (supplementary fig. S14, Supplementary Material online).

**Fig. 5.**
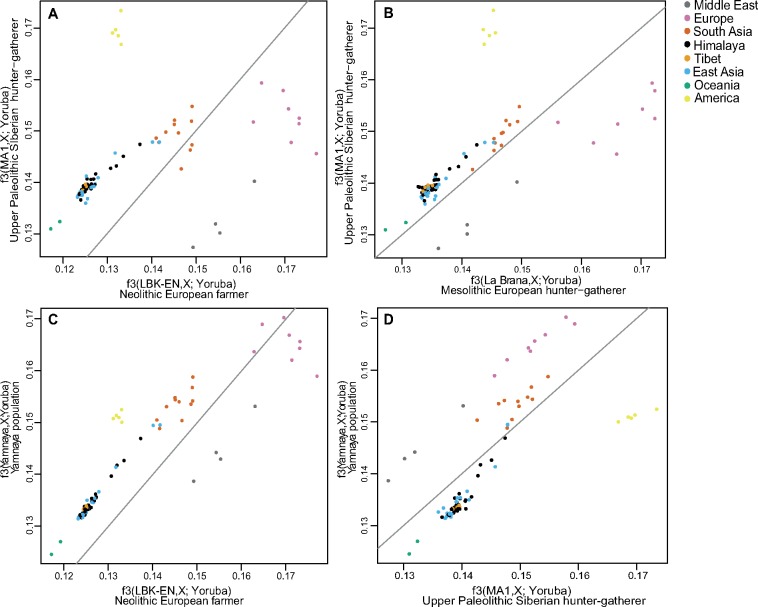
Relative genetic similarity of the Himalayan region and other populations to four ancient DNA samples. (*A–D*) Each plot shows a comparison between two ancient samples, and equal similarity is represented by the gray line. Each dot represents a present-day population. Thus, section (*A*) shows that the Himalayan region populations are more similar to the Upper Palaeolithic Siberian hunter-gatherer than to the Neolithic European farmer.

We explored the genetic affinity between the Himalayan populations and five ancient genomes using *f3*-outgroup statistics. Himalayans show greater affinity to Eurasian hunter-gatherers (MA-1, a 24,000-year-old Upper Palaeolithic Siberian), and the related Bronze Age Yamnaya, than to European farmers (5,500–4,800 years ago; fig. 5*A*) or to European hunter-gatherers (La Braña, 7,000 years ago; fig. 5*B*), like other South and East Asian populations. We further explored the affinity of Himalayan populations by comparing them with the 45,000-year-old Upper Palaeolithic hunter-gatherer (Ust’-Ishim) and each of MA-1, La Braña, or Yamnaya. Himalayan individuals cluster together with other East Asian populations and show equal distance from Ust’-Ishim and the other ancient genomes, probably because Ust’-Ishim belongs to a much earlier period of time (supplementary fig. S15, Supplementary Material online). We also explored genetic affinity between modern Himalayan populations and five ancient Himalayans (3,150–1,250 years old) from Nepal. The ancient individuals cluster together with modern Himalayan populations in a worldwide PCA (supplementary fig. S16, Supplementary Material online), and the *f3*-outgroup statistics show modern high-altitude populations have the closest affinity with these ancient Himalayans, suggesting that these ancient individuals could represent a proxy for the first populations residing in the region (supplementary fig. S17 and table S4, Supplementary Material online). Finally, we explored the genetic affinity of Himalayan samples with the archaic genomes of Denisovans and Neanderthals (Skoglund and Jakobsson 2011), and found that they show a similar sharing pattern with Denisovans and Neanderthals to the other South and East Asian populations. Individuals belonging to four Nepalese, one Cambodian, and three Chinese populations show the highest Denisovan sharing (after populations from Australia and Papua New Guinea) but these values are not significantly greater than other South and East Asian populations (supplementary figs. S18 and S19, Supplementary Material online).

### Signatures of Adaptation in the Himalayan Region

We searched for variants under positive selection within Himalayan populations living at high altitudes, using four approaches: 1) genome-wide Spearman’s correlation between derived allele frequency and altitude; 2) EMMAX, a genome-wide statistical test for association between SNP frequency and altitude that accounts for population substructure (Kang et al. 2010); 3) the Population Branch Statistic (PBS) which identifies SNPs with unusually high *F*_ST_ values between high- and low-altitude samples, compared with an outgroup population (Yi et al. 2010); and 4) BayEnv v2, a Bayesian framework for specifically testing association between allele frequency and environmental variables, such as altitude (Coop et al. 2010; Gunther and Coop 2013).

**Fig. 6.**
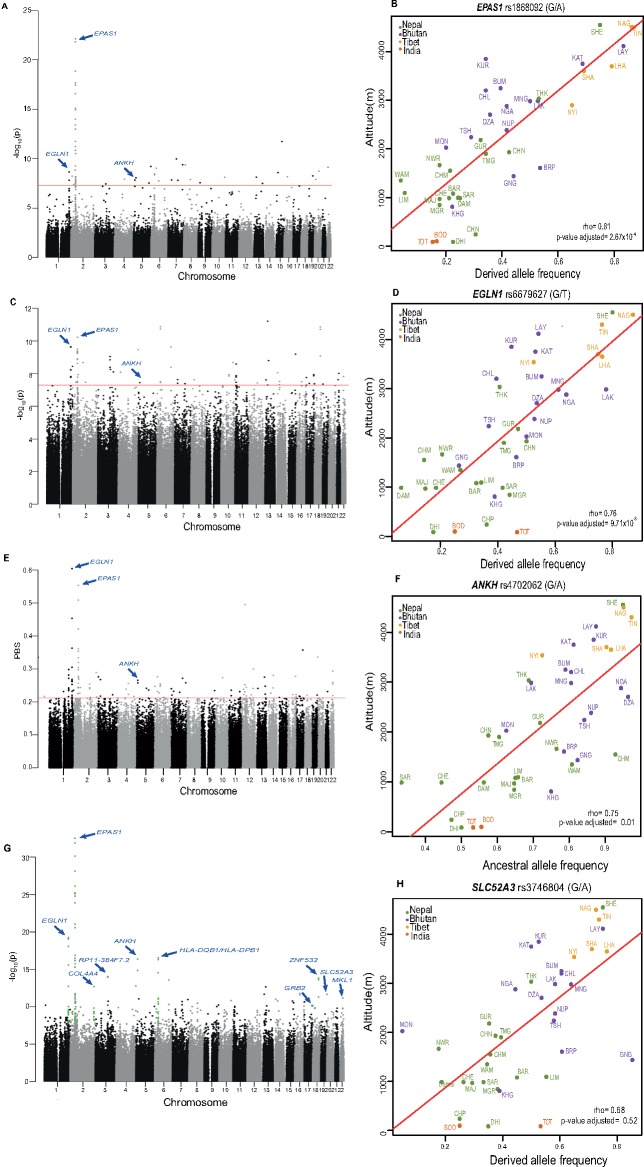
Signals of positive selection (adaptation) in the Himalayan populations. (*A*, *C*, *E*, *G*) Manhattan plots showing a measure of confidence in selection (vertical axis) plotted against genomic coordinate (horizontal axis). Each dot represents a SNP. (*A*) Spearman’s correlation between derived allele frequency and altitude. (*C*) EMMAX. (*E*) Population Branch Statistics. (*G*) Fisher’s combined *P* value from these three tests. (*B*, *D*, *F*, *H*) Plots of allele frequency against altitude for four selection candidates. Each dot represents a Himalayan region population. Population codes are defined in supplementary table S1, Supplementary Material online.

Genome-wide Spearman’s correlations pinpointed 75 derived alleles with frequencies that correlated significantly with altitude (Spearman’s *rho* >0.72) (fig. 6 and supplementary table S5, Supplementary Material online) while the EMMAX analysis showed that 99.98% of the variance was explained by the kinship matrix, but identified 56 variants where the observed allele frequency nevertheless diverged significantly from the expected frequency (fig. 6 and supplementary table S5, Supplementary Material online). The PBS analysis pinpointed 117 variants under possible selection for the derived allele, including ones in regions such as *EPAS1* and Disrupted in Schizophrenia 1 (*DISC1*) previously identified by Tibetan exome sequencing (Yi et al. 2010) (fig. 6 and supplementary table S5, Supplementary Material online).

Twelve candidate variants lying in three different genomic regions overlap between these first three approaches (fig. 6 and supplementary fig. S20 and table S5, Supplementary Material online). Ten of them lie on chromosome 2 in a ∼330-kb genomic region that includes *EPAS1*, of which two are of potential functional significance. These are rs1868092, downstream of *EPAS1* in a promoter-flanking region which has previously been associated with high-altitude adaptation and shown to be a single-tissue eQTL in whole blood (Petousi et al. 2014; Basang et al. 2015), and rs982414, an intronic variant ∼231 kb downstream of *EPAS1*, which has been associated with hemoglobin concentration in Tibetans (Beall et al. 2010). Furthermore, rs12986653, a variant in ATPase H+ Transporting V1 Subunit E2 (*ATP6V1E2*) which falls in a CTCF binding site, shows single-tissue eQTLs associated with the *ATP6V1E2*, CXXC repeat containing interactor of PDZ3 domain (*CRIPT*) and Transmembrane protein 247 (*TMEM247*) genes (The Genotype-Tissue Expression Consortium 2013) and has a high CADD score of 20.6 (supplementary figs. S21 and S22 and table S5, Supplementary Material online). The second region overlapping between the three methods is on chromosome 1 and includes the 11th candidate SNP, rs6679627, an intronic variant in the Tripartite Motif Containing 67 (*TRIM67*) near Egl-9 family hypoxia inducible factor 1 (*EGLN1*), which has previously been associated with high-altitude adaptation in Tibetans (Simonson et al. 2010; Lorenzo et al. 2014). The third overlapping region is on chromosome 5 and includes a SNP, rs4702062, which shows a strong EMMAX signal together with strong positive correlation with high altitude for the ancestral allele (between 80% and 97% in high-altitude populations, compared with a maximum frequency ≤64% in 1000 Genomes Project populations) and has a CADD score of 12.9 (fig. 6*F*). This variant is also in high-linkage disequilibrium LD (*r*^2^ = 0.87) with another nearby variant, rs844335, that was picked up by PBS because of its high derived allele frequency, between 80% and 97% in high-altitude populations, compared with a maximum frequency ≤61% in 1000 Genomes Project populations. rs4702062 lies in an intergenic region upstream of the ANKH inorganic pyrophosphate transport regulator (*ANKH*) gene on chromosome 5, while rs844335 lies within an open chromatin region nearby, and is also in LD (*r*^2^ = 0.73) with a third variant, rs1550825, that lies in a transcription factor binding site (supplementary fig. S23, Supplementary Material online). The *ANKH* gene codes for a transporter that regulates the passage of inorganic phosphate through the cell and contains two hypoxia-responsive elements (HREs) in proximity to its promoter region, and thus its expression is regulated by hypoxic factors (HIFs) (Zaka et al. 2009).

Combining the *P* values from the first three methods provides a concise way to merge their findings, although not a measure of the type-1 error rate because the tests are not completely independent. This approach identified 398 variants with Bonferroni-adjusted *P* value <0.01 (supplementary table S5, Supplementary Material online). The fourth method, BayEnv v2, could not be included in this combined *P* value analysis as it used an LD-pruned subset of the SNPs. The strongest signals of selection from this last analysis, with multiple significant SNPs in each, included the three regions surrounding *EPAS1, EGLN1*, and *ANKH* discussed earlier, and also a region near the major histocompatibility complex. The *EPAS1, EGLN1*, and *HLA-DQB1* regions were also reported as associated with high-altitude adaptation in a previous genome-wide association study between Tibetans and Han Chinese using a linear mixed model approach comparable to EMMAX (Yang et al. 2017). Multiple significant SNPs lying in these regions present single-tissue eQTLs and high CADD scores (supplementary table S5, Supplementary Material online). An additional six regions with two or more significant SNPs stood out in the combined *P* value analysis, surrounding the *RP11-384F7.2*, Zinc finger protein 532 (*ZNF532*), Collagen type IV alpha 4 chain (*COL4A4*), Solute carrier family 52 member 3 (*SLC52A3*), Megakaryoblastic leukemia (translocation) 1 (*MKL1*), and Growth factor receptor bound protein 2 (*GRB2*) genes (table 1 and fig. 6*G*). The results from BayEnv v2 were then used for further validation of the candidate genes highlighted above. It pinpointed 503 variants falling into the category “Decisive” [Bayes Factor (BF) >100, log_10_(BF) >2] (supplementary fig. S24 and table S5, Supplementary Material online). Eight of the top ten candidate regions discussed earlier overlapped with the “Decisive” ones: *EGLN1, EPAS1, COL4A4, RP11-384F7.2*, *ANKH, HLA-DQB1/HLA-DPB1, ZNF532*, and *SLC52A3* while the *MKL1* and *GBR2* regions were overlapped strong [10 < BF < 100, 1 < log_10_(BF)<2] and substantial [3.2< BF < 10, 0.5 < log_10_(BF)<1] candidates, respectively.

**Table 1.** Genomic Regions Showing the Strongest Signals of Positive Selection in the Himalayan Populations.

Candidate Gene	Cluster of Selected SNPs: GRCh37 Coordinates	Number of SNPs in Cluster	Top SNP: Combined *P* Value	Top SNP	Top SNP Frequency High-Altitude Populations	Top SNP Frequency East Asian Populations	Allele Under Selection	eQTLs	Comments
***EPAS1***	2: 46468276–46852033	26	1.83E-27	rs4953359	68%	14%	D	9	Known high-altitude selection signal
***EGLN1***	1: 231204794–231897303	21	3.45E-14	rs6655954	58%^a^	27%^a^	A	10	Known high-altitude selection signal
***HLA-DQB1/HLA-DPB1***	6: 32582075–33175824	15	1.66E-11	rs10484569	77%	39%	D	6	Known high-altitude selection signal. Region associated with susceptibility to HBV infection in East Asians (Guo et al. 2011)
***ANKH***	5: 14908578–149285033	5	2.63E-11	rs4702062	84%^a^	64%^a^	A	–	Novel: regulatory region. Gene has hypoxia responsive element (HREs) in promoter region and it has been reported as a candidate for high-altitude adaptation in Tibetan pigs (Ai et al. 2014)
***RP11-384F7.2 AC068633.1***	3: 117427214–118549344	5	6.68E-09	rs1081896	79%^a^	64%^a^	A	2	Novel
***ZNF532***	18: 56562356–56648324	3	8.37E-09	rs3826597	18%	8%	D	–	Novel
***COL4A4***	2: 227770592–227922321	8	1.19E-07	rs3769641	46%	19%	D	1	Novel: include synonymous and splice region variants. This gene encodes one of the subunits of collagen type IV. Collagen metabolism plays an important role in angiogenesis during hypoxia (Tajima et al. 2001; Sudhakar et al. 2005)
***SLC52A3***	20: 744415–745963	2	1.22E-06	rs3746804	63%	25%	D	1	Novel: missense and synonymous variants. Riboflavin transporter that could be involved in counteracting alterations of energetic metabolism under acute hypoxia (Ghosal et al. 2015)
*MKL1*	22: 40827319–40905072	3	3.97E-06	rs17001997	47%	24%	D	3	Novel: gene involved in the regulation of cellular response to hypoxia in the vasculature of rats (Yuan et al. 2014)
*GRB2*	17: 73326965–73374945	4	3.66E-05	rs4789182	94%	83%	D	4	Novel: associated with reduction of hypoxia-induced oxidative stress in Tibetan individuals (Li et al. 2016)

NOTE.—Bold candidate genes: Decisive (log_10_Bayes Factor >2) candidates from BayEnv v2.

a Frequency of ancestral allele.

A, Ancestral; D, Derived.

We highlight further features of these candidate regions. The *SLC52A3* region includes a missense variant (Pro267Leu, rs3746804) with derived allele frequency >70% in most high-altitude populations compared with a maximum frequency ≤35% in the 1000 Genomes Project populations, and a synonymous variant (rs3746807) with overall high derived allele frequency in Himalayan populations (42–100%) compared with a maximum frequency ≤24% in 1000 Genomes Project populations (fig. 6*H*). rs3746804 shows single-tissue eQTLs for *SLC52A3* in lung and skin, and has a CADD score of 13.3. The *COL4A4* region comprises eight SNPs: the top one, rs3769641, lies in a splicing regulatory region within *COL4A4*, and its derived allele frequency is positively correlated with altitude (Spearman’s *rho*= 0.70). This region also contains a missense variant (rs3752895) that shows single-tissue eQTLs in brain tissue for the Rhomboid domain containing 1 (*RHBDD1*) gene and a synonymous variant (rs2228557). These two variants show high CADD scores of 17.2 and 16.7, respectively. The *GRB2* region on chromosome 17 shows four intronic SNPs and has previously been associated with hypoxia-induced oxidative stress level at the intestinal mucosal barrier in Tibetans compared with Han Chinese (Li et al. 2016) (fig. 6*G*, table 1, and supplementary table S5, Supplementary Material online). Two of the four variants in *GRB2*, rs4542691 and rs4789182, show single-tissue eQTLs. The *MKL1* region on chromosome 22 carries three intronic SNPs, and has previously been associated with the regulation of the cellular response to chronic hypoxia in the vasculature of rats. All three variants in *MKL1*, rs2294352, rs6001931, and rs17001997, show single-tissue eQTLs in muscle-skeletal tissue (Yuan et al. 2014).

We also examined the allele frequencies of the top SNPs in our ten candidate regions (table 1) in the five ancient Himalayan genomes, and compared them with the allele frequencies in present-day Himalayans. Six variants in the *EPAS1* region and 11 in the *EGLN1* region show high derived allele frequencies in ancient Himalayans (≥ 0.60). The missense variant rs3746804 in the *SLC52A3* locus also shows a high derived allele frequency of 0.67 in the ancient Himalayans. Variants in *COL4A4, ANKH*, *RP11-384F7.2/AC068633.1*, and *HLA-DBP1/DBP2* show derived allele frequencies in the ancient Himalayans of 0.56–1.00, while two variants, rs4542691 and rs4789182, in the *GRB2* locus show a derived allele frequency of 100% in the ancient samples. Finally, rs3826597 in *ZNF532* region show a derived allele frequency of 0.95 in the ancient Himalayans (supplementary table S5, Supplementary Material online). None of the top selection candidate regions, apart from *EPAS1* (Huerta-Sanchez et al. 2014; Hu et al. 2017), show signatures of adaptive introgression from archaic Denisovans or Neanderthals according to published introgression maps (Sankararaman et al. 2016).

We also generated a protein homology model for SLC52A3, and investigated the position of the missense variant, rs3746804. The SLC52A3 structure resembles that of a glucose transporter and rs3746804 is predicted to lie in an exposed intracellular region which could act as an interaction surface for the intracellular environment (Iancu et al. 2013) (supplementary fig. S25, Supplementary Material online). We finally generated protein–protein interaction networks for our top ten protein candidates. *EPAS1, EGLN1, COL4A4*, and *GRB2* were predicted to be part of the same network. Prostaglandin I2 synthase (*PTGIS*) and Vitamin D receptor (*VDR*), suggested previously by Hu et al. to be under selection for high-altitude adaptation, are also predicted to be in the same protein–protein interaction network (Hu et al. 2017) (supplementary fig. S26, Supplementary Material online).

## Discussion

We have performed the most comprehensive survey thus far of genetic variation in the Himalayan region, aiming to elucidate the genetic ancestry of these populations, including their demographic histories, and the genetic adaptations they have undergone in order to survive in the varied and challenging environments present in the region.

### Population Structure and Demography

In the broadest sense, all the Himalayan populations share ancestry with their geographical neighbours in South and East Asia, reflecting the common pattern of the distribution of human genetic diversity dominated by geography (fig. 2*A*). Within this framework, we nevertheless detect an ancestral component that is abundant in most Himalayans, but rare elsewhere (fig. 2*C* with *K* values of 2–4; supplementary fig. S2, Supplementary Material online), pointing to shared ancestry for these populations, a conclusion reinforced by their similar patterns of shared genetic drift with non-Himalayan ancient samples (fig. 4). At finer resolution, we see evidence for both substructure reflecting geography within the Himalayan region, and extreme drift leading to single populations forming outliers in the PCA (fig. 2*B*) or specific components in ADMIXTURE analysis (fig. 2*C*). The most striking example is provided by the Toto from North India, an isolated tribal group with the lowest genetic diversity of the Himalayan populations examined here, indicated by the smallest long-term Ne (supplementary fig. S5, Supplementary Material online), and a reported census size of 321 in 1951 (Mitra 1951), although their numbers have subsequently increased. Despite this extreme substructure, shared common ancestry among the high-altitude populations (figs. 2*C* and 3) can be detected, and the Nepalese in general are distinguished from the Bhutanese and Tibetans (fig. 2*C*) and they also cluster separately (fig. 3). In a worldwide context, they share an ancestral component with South Asians (supplementary fig. S2, Supplementary Material online). On the other hand, the Tibetans do not show detectable population substructure, probably due to a much more recent split in comparison with the other populations (fig. 2*C* and supplementary fig. S6, Supplementary Material online). The genetic similarity between the high-altitude populations, including Tibetans, Sherpa, and Bhutanese, is also supported by their clustering together on the phylogenetic tree, the PCA generated from the coancestry matrix generated by fineSTRUCTURE (supplementary figs. S10 and S11, Supplementary Material online), the lack of statistical significance for most of the *D*-statistics tests (Yoruba, Han; high-altitude Himalayan 1, high-altitude Himalayan 2), and the absence of correlation between the increased genetic affinity to lowland East Asians and the spatial location of the Himalayan populations (supplementary figs. S12 and S13, Supplementary Material online). Together, these results suggest the presence of a single ancestral population carrying advantageous variants for high-altitude adaptation that separated from lowland East Asians, and then spread and diverged into different populations across the Himalayan region. Genetic drift and admixture with other Himalayan, South, and East Asian populations can explain the widespread distribution of the selected *EPAS1* haplotype at lower frequencies in populations at lower altitudes (Hackinger et al. 2016), and the altitude clines in the other selection candidates (fig. 6). Our findings suggest a recent split (only a proxy for population differentiation, given the limitations of the method applied) between Tibetans, Sherpa and, possibly, other high-altitude populations, rather than the Tibetans being a mixture of Sherpa and Han Chinese (Jeong et al. 2014; Bhandari et al. 2015). Whole-genome sequences from multiple high-altitude populations will provide better estimates of such divergence times and a more detailed demographic history of the region.

Himalayan populations show signatures of recent admixture events, mainly with South and East Asian populations as well as within the Himalayan region itself. Newar and Lhasa show the oldest signature of admixture, dated to between 2,000 and 1,000 years ago. Majhi and Dhimal display signatures of admixture within the last 1,000 years. Chetri and Bodo show the most recent admixture events, between 500 and 200 years ago (fig. 4 and supplementary table S3, Supplementary Material online). The comparison between the genetic tree and the linguistic association of each Himalayan population highlights the agreement between genetic and linguistic subdivisions, in particular in the Bhutanese and Tibetan populations. Nepalese populations show more variability, with genetic subclusters of populations belonging to different linguistic affiliations (fig. 3*B*). Modern high-altitude Himalayans show genetic affinity with ancient genomes from the same region (supplementary fig. S17, Supplementary Material online), providing additional support for the idea of an ancient high-altitude population that spread across the Himalayan region and subsequently diverged into several of the present-day populations. Furthermore, Himalayan populations show a similar pattern of allele sharing with Denisovans as other South-East Asian populations (supplementary figs. S18 and S19, Supplementary Material online). Overall, geographical isolation, genetic drift, admixture with neighbouring populations and linguistic subdivision played important roles in shaping the genetic variability we see in the Himalayan region today.

### High-Altitude Adaptation

The harsh environment at high altitude due to increased ultraviolet radiation, hypobaria and hypoxemia is inescapable, so it is expected to have triggered physiological and genetic adaptations including modifications in the cellular responses of the humans who settled there. Genomic scans for positive selection in Tibetans have previously implicated several genes as candidates for high-altitude adaptation, especially an extended *EPAS1* haplotype (Yi et al. 2010; Peng et al. 2011, 2017; Xu et al. 2011; Lu et al. 2016) that arose by introgression from Denisovans (Huerta-Sanchez et al. 2014), and is widespread in the region (Hackinger et al. 2016). Positive selection scans can easily be confounded by population structure, and although simple correlations of SNP frequency with altitude replicated several of the candidates reported in previous studies (fig. 6 and supplementary table S5, Supplementary Material online), including those near *EPAS1*, *DISC1*, and *ATP6V1E2* which are highly differentiated between lowland Han and Tibetans (Yi et al. 2010; Lu et al. 2016), additional analyses better suited to substructured populations only confirmed a subset of these. The signal on chromosome 2 is particularly strong and includes a ∼330-kb region encompassing *EPAS1, ATP6V1E2*, and *PIGF/CRIPT* (fig. 6*B* and *C* and supplementary figs. S21 and S22, Supplementary Material online). An expected signal of selection from *EGLN1* was observed via nearby variants in *TRIM67* and *TSNAX-DISC1* (fig. 5*D*) (Xiang et al. 2013; Foll et al. 2014). A novel signal of selection was found in the region upstream of *ANKH* on chromosome 5 (fig. 6*F*). This region shows extended LD, but the variant driving the selection could not be identified by our analysis (supplementary fig. S23, Supplementary Material online). Nevertheless, *ANKH* is itself a strong candidate because it is involved in the regulation of the transportation of inorganic phosphate and its expression is regulated by HIF2A (*EPAS1*) and HIF1A (Zaka et al. 2009; Skubutyte et al. 2010). ANKH is essential for maintaining cellular function and bone mineralization, and its concentration plays a central role in several metabolic pathways (Dick et al. 2011).

In order to maximize the power to identify the additional selection candidates, we calculated combined *P* values for three different statistics applied to our data set, and then further validated these candidate genomic regions using a fourth statistic, BayEnv2. (fig. 6*G*, table 1, and supplementary fig. S24 and table S5, Supplementary Material online). Some of these additional variants may play important roles in the hypoxic environment, contributing to physiological responses to hypoxia. *COL4A4* encodes one of the subunits of collagen type IV, which is an essential component of basement membranes, and plays an important role in angiogenesis. Hypoxia exposure triggers vasoconstriction which requires structural remodelling of arterial vessels, especially in lung, and collagen metabolism is required for this process (Tajima et al. 2001; Sudhakar et al. 2005). *GRB2* is involved in the regulation of reactive oxygen species (ROS) production in hypoxic environments and it has been shown that, in Tibetans, downregulation of its expression reduces ROS damage and improves glucose and fat metabolism in intestinal tissues (Li et al. 2016). *MKL1* encodes a myocardin-related transcription factor and is involved in smooth muscle cell differentiation (Cen et al. 2003). Down-regulation of *MKL1* reduces the pulmonary arterial pressure in response to chronic hypoxia and regulates vascular remodelling in rats (Yuan et al. 2014). *SLC52A3* encodes a transporter of riboflavin, a vitamin that modulates fatty acid and amino acid metabolism and reduces cellular oxidative stress (Ghosal et al. 2015). Riboflavin supplementation of the diets of mice improves their energetic metabolism under acute hypoxia; Thus, increased riboflavin could be effective in counteracting the alteration of human metabolism in hypoxic conditions (Wang et al. 2014). SLC52A3 is a transmembrane protein and the homology-based protein model we generated resembles the structure of a glucose transporter; our top candidate variant, rs3746804 (Pro267Leu), lies in the intracellular environment in a possible interaction region of the protein surface (supplementary fig. S25, Supplementary Material online). This selection signal seems to be specific for Himalayan populations and could be related to the diet and environment, where efficient intake of riboflavin at high altitude would be advantageous (Blanck et al. 2002). Two out of three additional candidates for high-altitude adaptation (*PTGIS* and *VDR*) suggested by Hu *et al.* are predicted to be in the same protein–protein interaction pathway as some of our candidates, *COL4A4* and *GRB2*, and linked with other genes (*EPAS1, EGLN1, HIF1A, VHL*) involved in the hypoxic response (supplementary fig. S26, Supplementary Material online) (Hu et al. 2017). *ANKH* has also been reported as a candidate for high-altitude adaptation in Tibetan pigs (Ai et al. 2014).

Thus, of the top ten selected candidate regions (seven novel) highlighted by our work (table 1), four are members of the most relevant protein–protein interaction network and three others have known functions relevant to high-altitude adaptation: findings that are very unlikely due to chance. Furthermore, despite the strong ascertainment bias of the SNPs included on SNP-chips, variants lying in our top ten candidate regions are associated with single-tissue eQTLs and present high CADD scores, suggesting their possible importance in gene regulation and expression. The presence of high derived allele frequencies of variants in *EGLN1*, *EPAS1*, *SLC52A3*, and *GRB2* loci in ancient Himalayans also supports our hypothesis that these candidates may be under selection and important for high-altitude adaptation (supplementary table S5, Supplementary Material online). According to available introgression maps (Sankararaman et al. 2016), none of the top selected candidate regions, apart from the well-known *EPAS1* intronic region (Huerta-Sanchez et al. 2014; Hu et al. 2017), show signatures of adaptive introgression from Denisovans or Neanderthals. In all cases, high-coverage whole-genome sequences and comparisons with other species that have adapted to similar environments should help to identify or confirm the key causal variants and suggest strategies for functional follow-up.

In conclusion, the current analyses have established the broad features of Himalayan genetic variation: a South or East Asian substrate influenced by local differentiation and mixing in ways that are now understood in outline, including extreme genetic drift in several populations. It has provided a comprehensive data set from the region for the community to use in future studies. In addition, there is evidence for early strong genetic adaptation to high-altitude living followed by spread of the adapted population. Future functional investigations will allow these phenomena to be understood in more detail.

## Materials and Methods

### Samples

Eight hundred and eighty-three individuals belonging to 49 Himalayan populations were genotyped and analyzed after obtaining informed consent. The data set included 26 populations from Nepal, 16 from Bhutan, two from North India sampled in Bhutan, and five from Tibet in China (fig. 1 and supplementary table S1, Supplementary Material online). The samples represent the two major linguistic families in the area: Indo-European and Tibeto-Burman (also known as Trans-Himalayan). Specifically, 44 populations comprise Tibeto-Burman speakers from Tibet, Bhutan, North India, or Nepal, and five comprise Indo-European speakers from Nepal (Chetri, Damai, Majhi, Sarki, and Sonar). The Bhutanese, North Indian, and Nepalese samples were collected as part of the “Language and Genes of the Greater Himalayan Region” project, a genetic survey of Tibeto-Burman and Indo-European speakers from these Himalayan countries, and have been described previously (Kraaijenbrink et al. 2014). Tibetan samples were selected from participating members of an epidemiological study in the Tibet Autonomous Region, China, in 2007 that was approved by the institutional ethics review board of BGI-Shenzhen. Samples were collected from healthy unrelated Tibetans from five villages based on their medical records and a comprehensive medical examination during sampling. Peripheral venous blood or saliva was collected for DNA extraction and genotyping. All participants had a self-reported family history of at least three generations living at the sampling site.

### Genotyping and Quality Control

The samples were genotyped using three Illumina SNP-chips: 1) HumanOmniExpress-12 v1.0 Bead Chip (741,000 SNPs) at the Wellcome Sanger Institute; 2) HumanOmni1-Quad BeadChip (∼1 M SNPs) at the Leiden University Medical Center; and 3) HumanOmniExpress-24 BeadChip (∼713,000 SNPs) at BGI-Shenzhen. Genotype calling and QC on all samples were performed using the Sanger Institute’s variant calling pipelines, and SNP positions were mapped to the human reference assembly GRCh37. Genotypes from the three arrays were merged using PLINK 1.92 (Purcell et al. 2007), resulting in a data set of 600,838 SNPs. Genotyping success rate and sample missingness thresholds were set to 99% and 10%, respectively. Sex-linked and mitochondrial SNPs as well as autosomal ones with Hardy–Weinberg Equilibrium *P* value <0.0000001 were removed. We also removed related samples (PI_HAT > 0.35) and outliers using EIGENSOFT 6.0 (Patterson et al. 2006; Price et al. 2006). These filters resulted in a final data set of 738 individuals and 583,011 SNPs. For comparison with worldwide populations, the Himalayans were merged with published data sets (supplementary table S1, Supplementary Material online) (Li et al. 2008; Chaubey et al. 2011; Metspalu et al. 2011) resulting in 1,962 individuals and 268,861 SNPs. Two additionally pruned data sets were generated from this by filtering out SNPs in high LD (*r*^2^ > 0.5). The pruned Himalayan data set consisted of 256,506 SNPs, and the pruned worldwide data set included 190,287 SNPs. For comparison with ancient samples, we generated two further data sets: 1) we merged our data with the Human Origins data, a data set comprising both modern and ancient individuals including archaic genomes from Denisovans and Neanderthals, and a chimpanzee (Patterson et al. 2012), resulting in 82,647 SNPs in common; and 2) we merged our Himalayan and worldwide data sets with published ancient Himalayan genomes from the Annapurna Conservation Area in Nepal (Jeong et al. 2016). From the published ancient BAMs, we randomly sampled (Korneliussen et al. 2014) a single sequence with a minimum quality of ≥20 to represent each SNP in our Himalayan data set, trimming 5 bp from both ends of reads to reduce the effect of ancient DNA deamination. This resulted in 582,810 SNPs in our Himalayan data set being covered by at least one of the ancient samples (supplementary table S1, Supplementary Material online).

### Population Characterization and Demography

The genetic structure of the Himalayan populations was examined using several statistical approaches. Principal component analysis (PCA) using EIGENSOFT 6.0 was performed using the LD-pruned data sets. For the worldwide data set, the eigenvectors were calculated using the global diversity and the Himalayan individuals were projected onto the plot (fig. 2). ADMIXTURE v1.2 (Alexander et al. 2009) was used on the pruned data sets for cluster analysis and the cross validation (CV) error for identifying the best *K* value. Estimation of long-term effective population size (Ne) for each Himalayan population and population divergence time was performed using the NeON R package (Mezzavilla and Ghirotto 2015), which calculates the harmonic mean of the population size at each generation and the time of divergence between populations in generations. More specifically, using LD information (*r*^2^) and recombination distance (*c*) we estimated the effective population size using the nonlinear regression model: *y_i_* =1/(α + β*c_i_*)+*e_i_*, with *y_i_* = (*r*^2^ 1/*n*) (*r*^2^ adjusted for chromosome sample size) for SNP pair *i* at recombination distance *c_i_* (in Morgans). We estimated the change in population size over time, as LD between loci with a recombination rate of *c* that reflects the ancestral effective population size 1/(2*c*) generations ago (Hayes et al. 2003). The model is based on the assumption of linear growth/decline. However, some populations might depart from the assumed model characteristics and LD patterns in these will be affected, so the relationship t = 1/(2*c*) should be viewed only as an approximate but useful indication of timeframes (de Roos et al. 2008). Furthermore, the time of divergence estimates are based on the assumption there was a “clean” population split, and migration will create a stronger correlation of LD (larger values of *r*^2^), thereby biasing the estimate of divergence time downward. Nevertheless, this method is still useful to assess isolation and difference in Ne between populations (McEvoy et al. 2011; Tassi et al. 2015). Only populations with sample size ≥10 were used, as the harmonic mean is sensitive to sample size. For all analyses, we assumed a generation time of 29 years (Fenner 2005). ROHs were identified using PLINK 1.92 (Purcell et al. 2007) with specific thresholds to maximize the detection of autozygous segments in the Himalayan populations (Howrigan et al. 2011) and other worldwide populations: a pruned data set (LD, *r*^2^ > 0.5) with only common variants (MAF >0.05) was used. The minimum number of SNPs to call an ROH was set to 100, the heterozygote allowance was set up to zero, the missing SNP allowance was set to 5 (5% of SNP threshold), and the window threshold to call an ROH was set to 0.05. The coefficient of inbreeding (F) was calculated with PLINK (Purcell et al. 2007).

A worldwide data set using a maximum of ten individuals from every population was used in the ChromoPainter and fineSTRUCTURE-2.0.6 (Lawson et al. 2012) analyses. The haplotypes were phased using SHAPEIT (Delaneau et al. 2011) using the 1000 Genomes Project Phase 3 (The 1000 Genomes Project Consortium 2015) as a reference panel. ChromoPainter infers the ancestry of each individual by reconstructing their haplotype segments from other individuals in the data set. FineSTRUCTURE uses the coancestry matrix inferred from ChromoPainter to construct a population-relationship tree and was run with 10,000,000 burn-in steps and 10,000,000 iterations. A PCA was also performed using the coancestry matrix generated by fineSTRUCTURE.

To assess the robustness of the results from the above data set, we additionally ran fineSTRUCTURE on a data set with fewer samples (the Himalayans and 1000 Genomes Project Phase 3 populations) but more markers (579,640 SNPs) using the same parameters. To test whether or not genetic similarity among high-altitude populations correlates with their geographical location, we used YRI (Yoruba in Ibadan, Nigeria) as an outgroup and calculated *D*-statistics (qpDstat function in ADMIXTOOLS v3 package) using the following phylogeny: *D*(Yoruba, Han; high-altitude Himalayan 1, high-altitude Himalayan 2) where high-altitude Himalayan 1 and high-altitude Himalayan 2 are pairs of Sherpa, Tibetan, or Bhutanese populations from an altitude of 2,500 m or above sea level (Patterson et al. 2012; Jeong et al. 2017). We computed *D*-statistics with the above phylogeny using our worldwide data set (supplementary tables S1 and S3, Supplementary Material online). Then, we tested the correlation between values of *D*-statistics with pairwise differences in longitude and latitude for each pair of populations using the Mantel test implemented in the “Ade4” R package (mantel.rtest function) (Dray and Dufour 2007).

Population admixture was studied using ALDER v1.03 (Loh et al. 2013), three-population statistics (*f3*) (Reich et al. 2009; Patterson et al. 2012), and TreeMix 1.12 (Pickrell and Pritchard 2012). Only populations with at least six individuals were included in these tests. ALDER was used with the default parameters and the threshold of LD in the reference groups was inferred by the program. A test was considered positive when both the 2-ref weighted LD curve was significant and the decay rates between the 2-ref and 1-ref curves were consistent. We considered a jack-knife block of 500 SNPs for *f3* statistic analyses. Shared genetic drift between modern populations and ancient samples was tested using outgroup *f3* statistics (ancient genome, X, Yoruba) (Patterson et al. 2012) with Yoruba as an outgroup. The ancient genomes used in this investigation were: 1) Eurasian hunter-gatherer (MA1,24,000-year-old Upper Palaeolithic Siberian) (Raghavan et al. 2014); 2) Bronze Age Yamnaya population (3,500–2,700 year old) (Allentoft et al. 2015); 3) Neolithic European farmer (LBK_EN, 5,500–4,800 year old) (Haak et al. 2015); 4) Mesolithic hunter-gatherer (La Braña, 7,000 year old) (Olalde et al. 2014); 5) Eurasian hunter-gatherer (Ust’-Ishim, 45,000 year old Upper Palaeolithic Siberian) (Fu et al. 2014); 6) five ancient Himalayan genomes 3,150–1,250 years old (C1, M63, S10, S35, and S41) from the Annapurna Conservation Area, Nepal (Jeong et al. 2016). We also used the archaic Denisovan and Neanderthal genomes and the chimpanzee to study genetic affinity of Himalayan samples to these archaic individuals: we calculated principal components using Denisovan, Neanderthal, and chimpanzee, and projected modern samples onto them (Skoglund and Jakobsson 2011). We also computed *D-*statistics (Yoruba, X; Denisovan, Chimpanzee) where X are different modern human populations from the worldwide data set).

### Positive Selection

Signals of positive selection were evaluated in four ways. First, we considered the Spearman correlation between derived allele frequency and the residence altitude of each population (Hackinger et al. 2016), adjusting the *P* value for multiple tests by applying the Bonferroni correction (requiring <0.05/number of tests). Second, we calculated a genome-wide association between allele frequency and altitude using a mixed model approach implemented in the Efficient Mixed-Model Association eXpedited program (EMMAX) (Kang et al. 2010). EMMAX detects variants where the observed allele frequency is significantly divergent from the expected frequency, and accounts for population stratification and sample relatedness through a variance component approach. A kinship matrix was constructed to account for population structure and implemented in a linear mixed model. Variants with *P* value <5×10^−8^ were considered significantly associated with altitude. Although these methods detect associations between allele frequency and altitude, they are not able to distinguish between high- and low-altitude selection signals. Third, we also calculated the Fixation Index (*F*_ST_) (Reynolds et al. 1983) for each SNP position between Himalayan, European (CEU; Utah Residents [CEPH] with Northern and Western European Ancestry) and East Asian (CHB; Han Chinese in Beijing, China) from the 1000 Genomes Project Phase 3 populations, and searched for unusual values using the Population Branch Statistic (PBS) (Yi et al. 2010). To reduce noise due to population structure within the Himalayan populations and differences in sample sizes, we ran PBS following the approach described (Yi et al. 2010), assuming that the CHB is the most closely related population to Tibetans and looked specifically for signals of high-altitude adaptation (Yi et al. 2010). For this analysis, we only used the populations from Bhutan and Tibet that clustered together in the fineSTRUCTURE analysis compared with CEU and CHB. Variants above the 99.99th percentile of the empirical distribution were considered statistically significant (Ayub et al. 2015; Fumagalli et al. 2015). The top hits from each method were assessed and the overlap collated. LD estimations for the regions containing the top candidates were calculated and plotted using Haploview (Barrett et al. 2005). We also used Fisher’s method (Fisher 1950) for combining *P* values of the three statistics used for detecting positive selection: 1) we calculated a rank *P* value of the PBS values (values were ranked in decreasing order from the most significant value and divided by the total number of SNPs used in the analysis); 2) we combined the *P* values of the three statistics genome-wide; and 3) we adjusted the *P* value for multiple tests by applying the Bonferroni correction (requiring < 0.01/number of tests). Finally, to further validate our selection signals, we calculated genome-wide associations between allele frequency and altitude using BayEnv v2 (Coop et al. 2010; Gunther and Coop 2013), a Bayesian framework specifically designed to detect correlation between allele frequencies and environmental factors taking population structure into account. The input files for BayEnv2 v2 were generated from a LD (*r*^2^ > 0.5) pruned SNP file using PGDSpider (Lischer and Excoffier 2012) and altitude (the environmental variable) was standardized according to the BayEnv2 v2 manual. BayEnv v2 was run with the default parameters and the Bayes Factors interpreted according to previous recommendations (Kass and Raftery 1995): only candidate variants falling into the category “Decisive” [Bayes Factors (BF) > 100, log_10_(BF) > 2] were considered significant. Where possible, we also calculated allele frequencies in the five Himalayan ancient genomes for our top candidates of selection.

We generated a protein homology model for SLC52A3 using Phyre2 software (Kelley et al. 2015) and mapped the missense variant found in *SLC52A3* onto the protein structure using PyMOL (The PyMOL Molecular Graphics System, Version 1.8 Schrödinger, LLC). We predicted protein–protein interaction networks using the STRING software (v. 10.5) (Szklarczyk et al. 2015) for our top selection candidates. Finally, we used the Ensembl Variant Effect Predictor (VEP) (McLaren et al. 2016) to predict the consequences of variants of interest on gene expression and protein sequence. We retrieved the Combined Annotation Dependent Depletion v.1.2 (CADD) scores (Kircher et al. 2014) of our top candidates and also overlapped our results with the Genotype-Tissue Expression (GTEx) database (The Genotype-Tissue Expression Consortium 2013). 

## Data Availability

All the genotype data are available from European Genome-phenome Archive under accession number EGAS00001002731.

## Web Resources

Ensembl VEP: http://grch37.ensembl.org/info/docs/tools/vep/index.html, last accessed May 10, 2018

GTEx Portal: https://www.gtexportal.org, last accessed May 10, 2018

Phyre2: http://www.sbg.bio.ic.ac.uk/phyre2, last accessed May 10, 2018

PLINK: https://www.cog-genomics.org/plink2, last accessed May 10, 2018

PyMOL: https://www.pymol.org/, last accessed May 10, 2018

STRING: http://string-db.org, last accessed May 10, 2018

Combined Annotation Dependent Depletion (CADD): http://cadd.gs.washington.edu/, last accessed May 10, 2018

## Supplementary Material

Supplementary data are available at *Molecular Biology and Evolution* online.

## Supplementary Material

Supplementary Data
