# Supplementary material for: Demographic History and Genetic Adaptation in the Himalayan Region Inferred from Genome-Wide SNP Genotypes of 49 Populations
Source: Mol Biol Evol. 2018 May 22;35(8):1916–33. doi: 10.1093/molbev/msy094 (PMC6063301; doi:10.1093/molbev/msy094)
Supplement: Supplementary Data [file msy094_supp.zip › Arciero-rev2_Supplementary_figures.pdf]

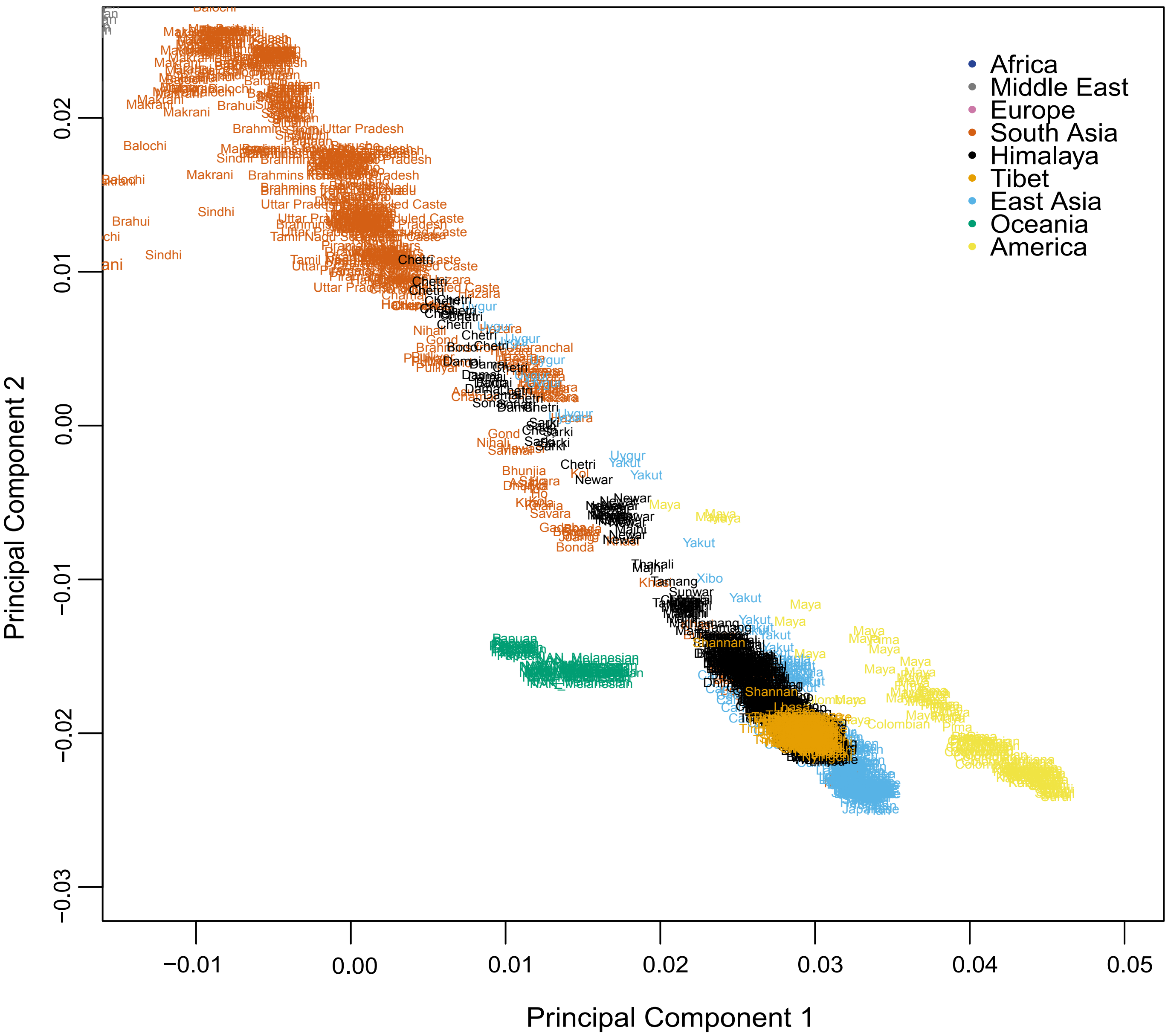

Fig. S1. Zoom of PCA using the world dataset. The plot displays a zoom of Himalayan populations in their worldwide context. Nepalese samples lie close to Indians, whereas Bhutanese and Tibetans are close to East Asian populations.

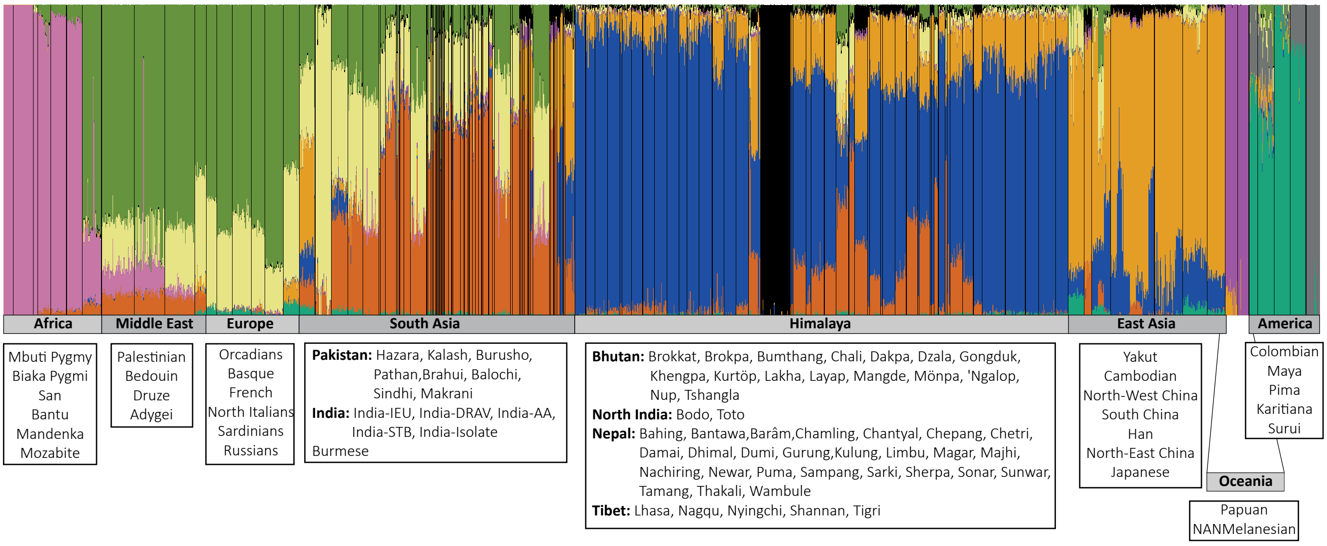

Fig. S2. ADMIXTURE analysis using the world dataset. The plot shows ADMIXTURE results for K=10. Himalayan populations display their own ancestral component (blue) that is also visible in some of the Pakistani and East Asian populations. Himalayan populations are also characterised by the presence of South Asian (orange) and East Asian (gold) components.

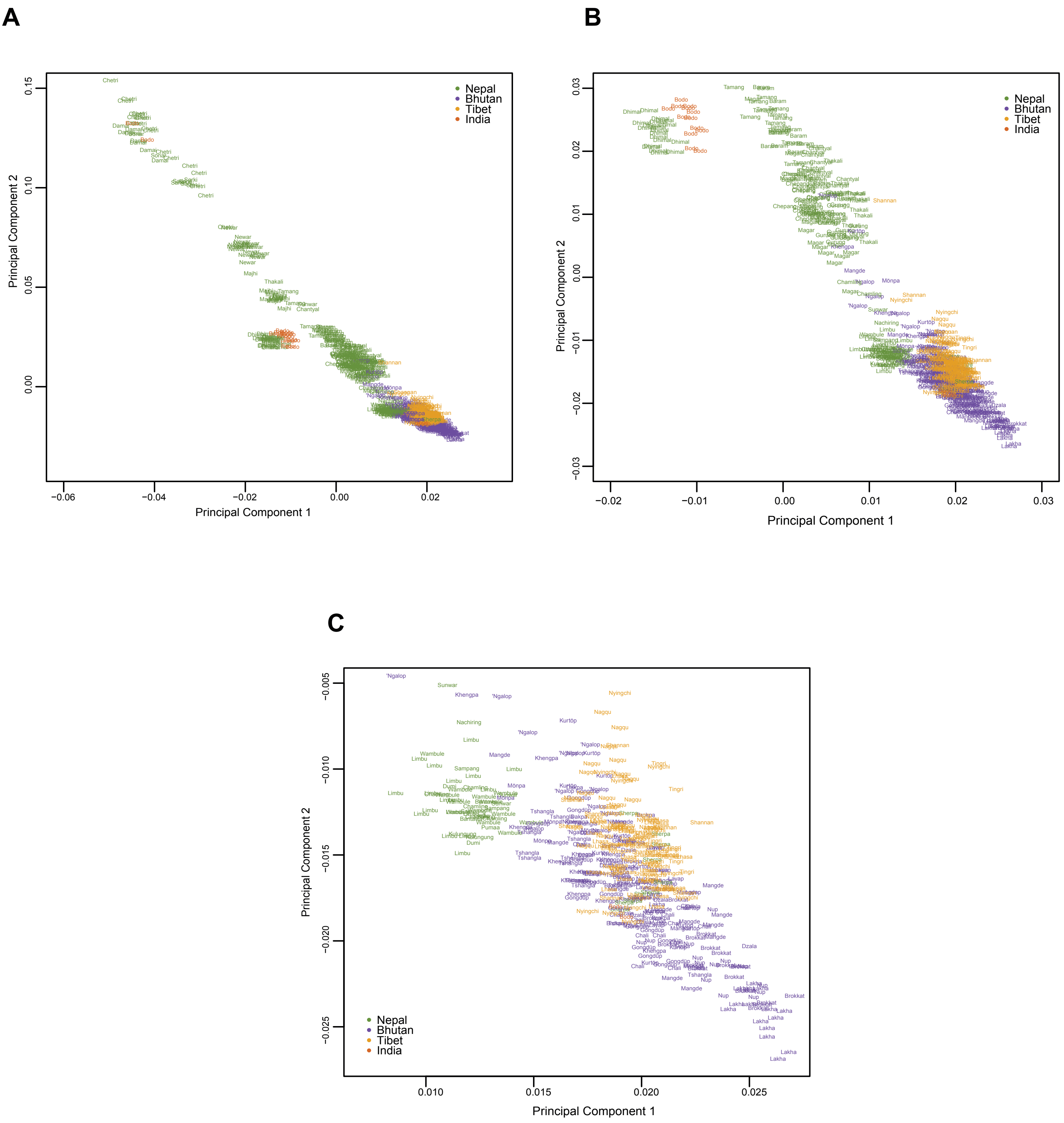

Fig. S3. Zoom of PCA using the Himalayan dataset only. A, B. The plot displays the Himalayan population substructure with Nepalese samples forming different clusters and Bhutanese and Tibetans clustering together. One separate cluster comprises the Dhimal and Bodo populations. C. The plot displays Bhutanese and Tibetans clustering together. Sherpa from Nepal cluster with other high-altitude populations from Bhutan and Tibet.

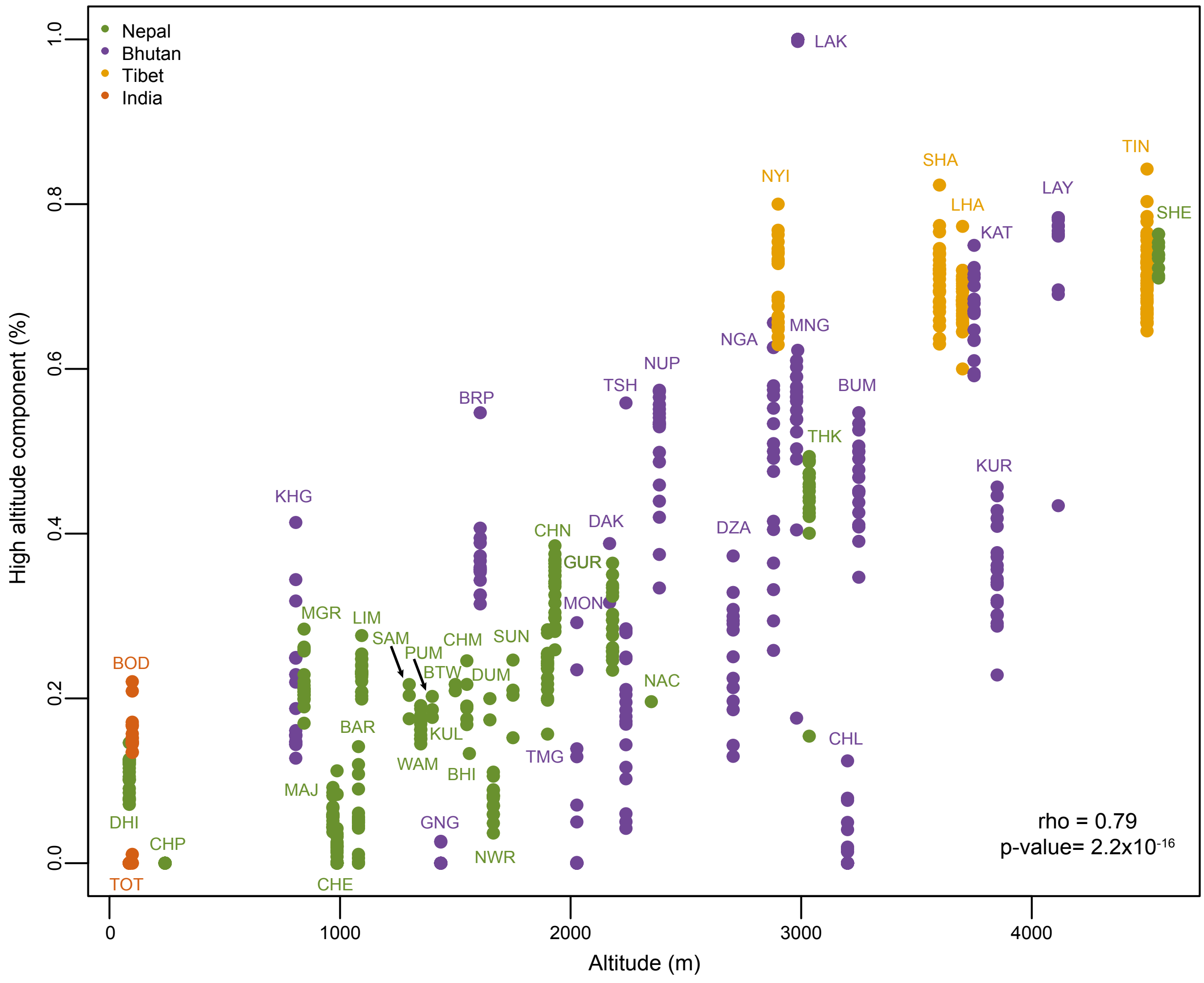

Fig. S4. Positive correlation between the high-altitude-specific genetic component and altitude. The plot shows the correlation between the percentage of the grey component from the ADMIXTURE analysis (K=6) in each Himalayan population (Figure 2c) and the altitude at which the population resides.

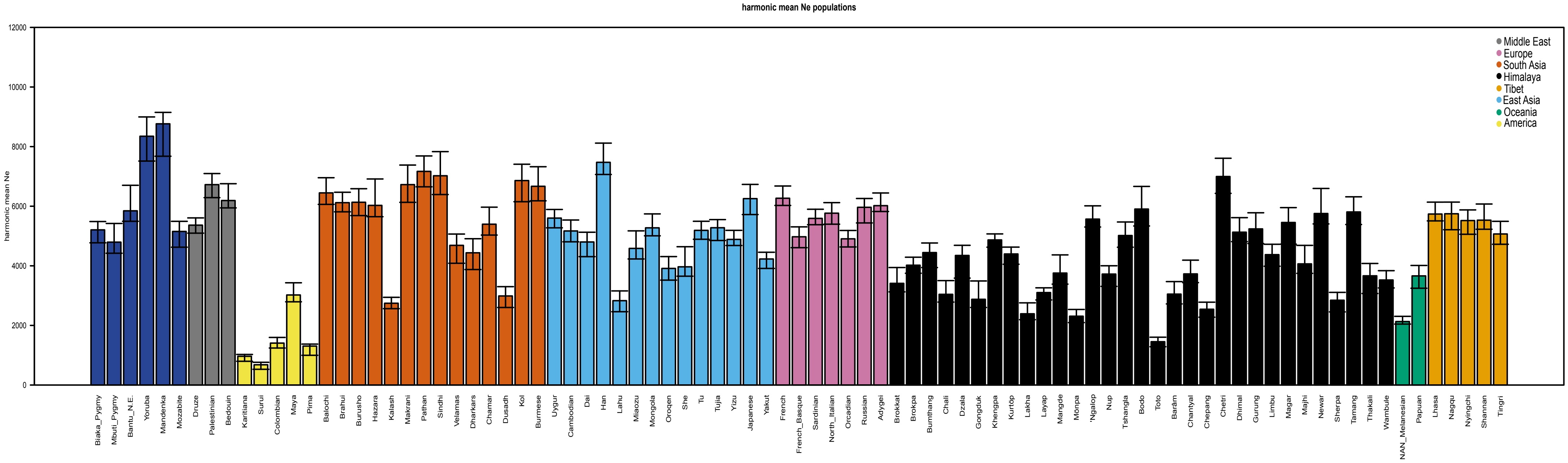

Fig. S5. Long-term effective population size (Ne) of Himalayan populations. The plot displays the harmonic mean of the Ne for each population. Only populations with sample size ≥ 10 were used for this analysis. Within the Himalayan populations, Chetri have the highest Ne, whereas Toto have the lowest.

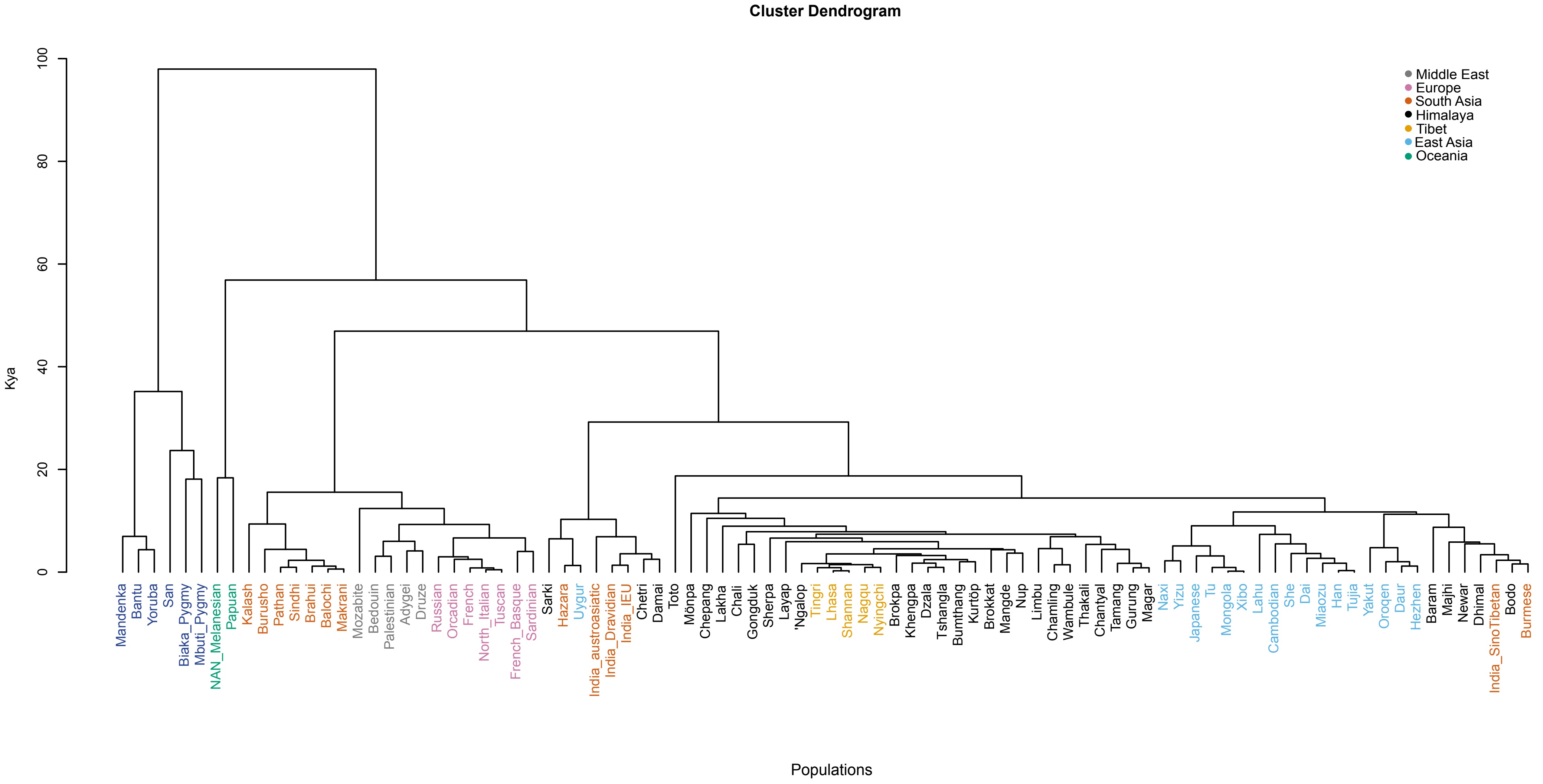

Fig. S6. UPGMA tree based on estimated time of divergence of the Himalayan populations. On the x-axis are represented the populations analysed and on the y-axis the time of divergence (Kya). Most of the Himalayan populations show a split time with Indian populations around 25,000-20,000 years ago and with East Asians around 15,000-10,000 years ago. Chetri, Damai and Sarkí are outliers to other Himalayan populations as they show divergence times similar to Indians. Tibetan populations display a split time among them of around 1,000 years ago.

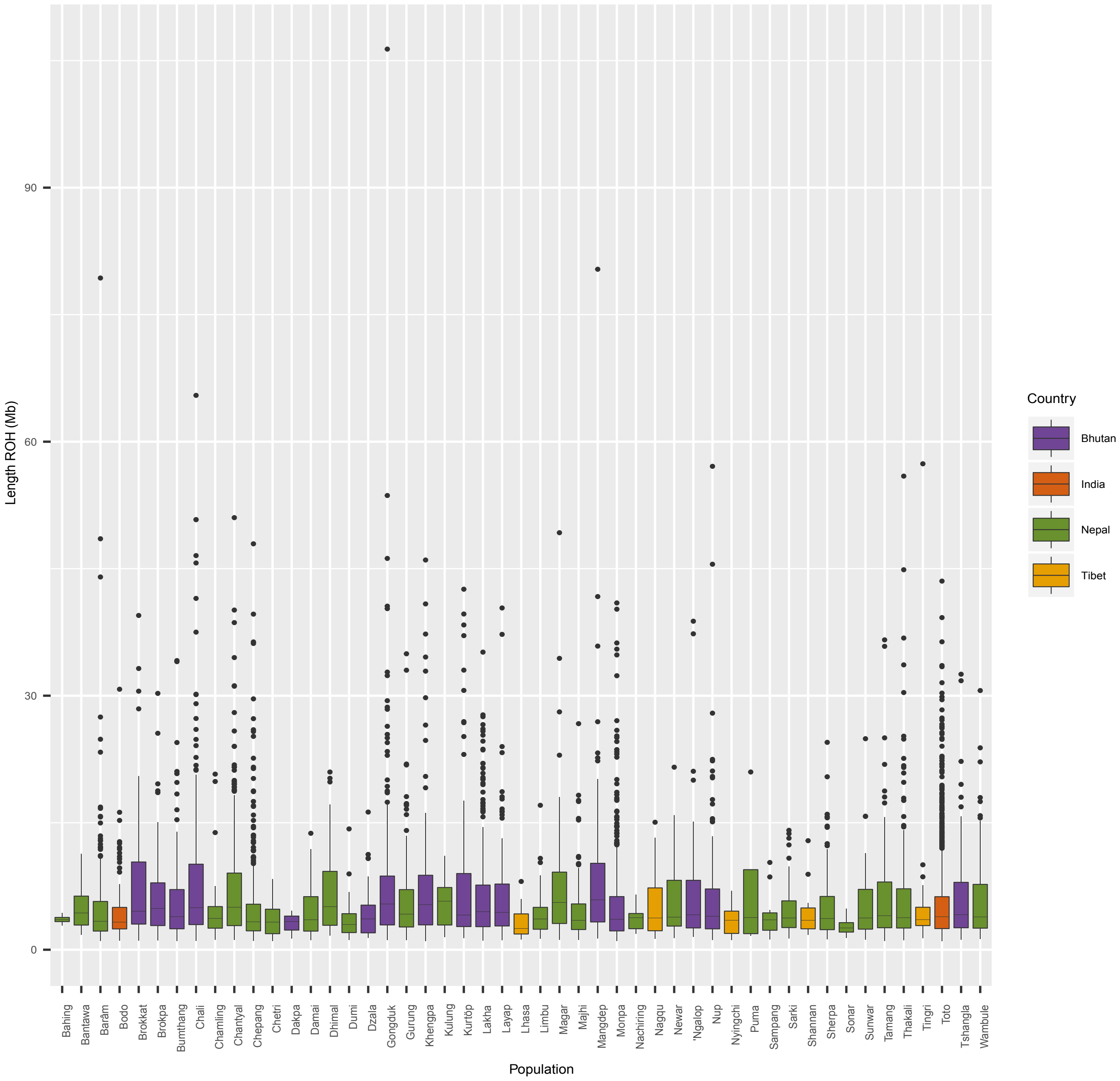

Fig. S7. Runs of homozygosity (ROHs) in the Himalayan populations. The x-axis reports the populations considered in the analysis and the y-axis represents the median length of ROHs in each population. Nepalese and Bhutanese populations show the highest number and longest ROHs, whereas Tibetan populations show the lowest number and length of ROHs.

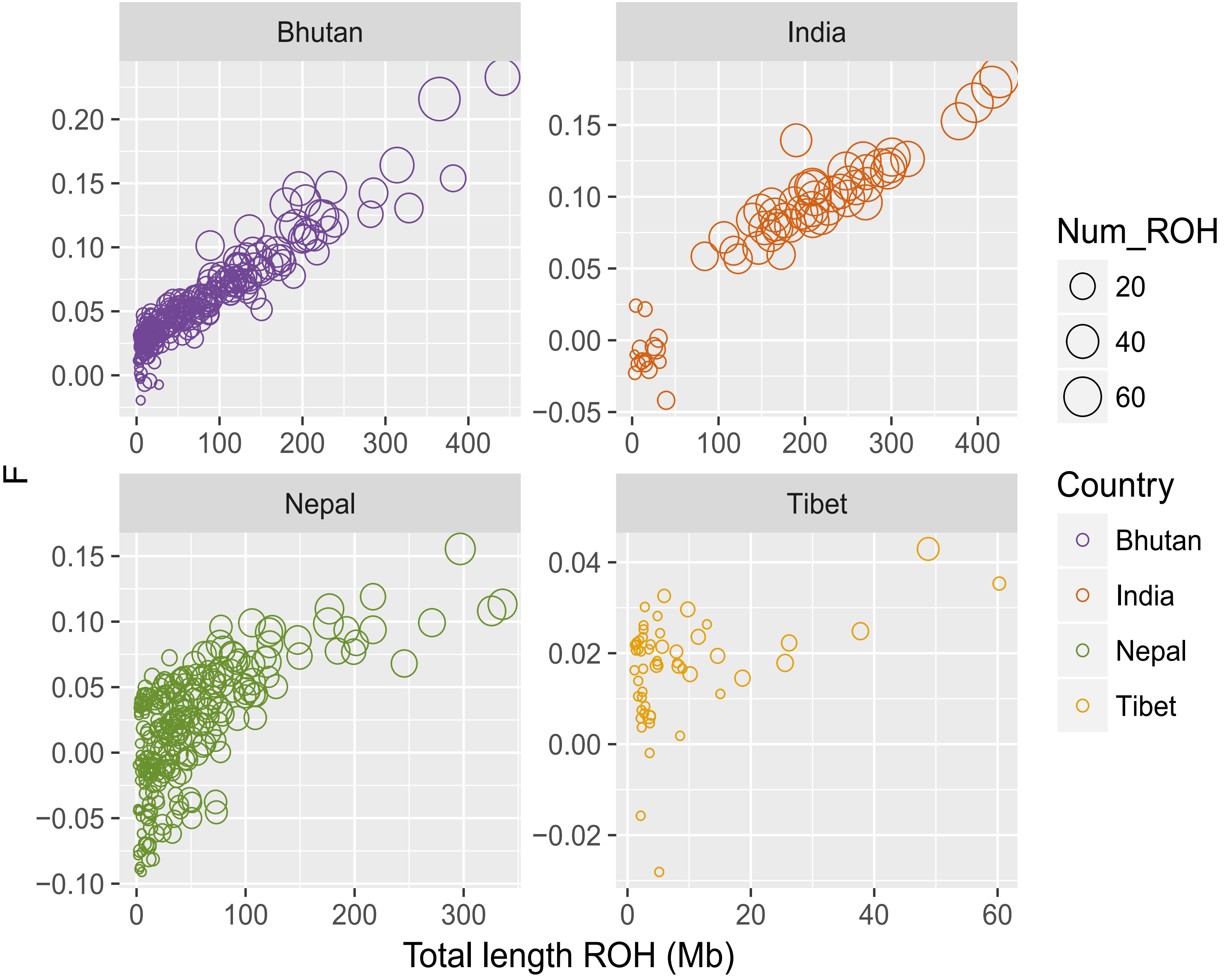

Fig. S8. Positive correlation between total length of ROHs per sample and coefficient of inbreeding (F). On the x-axis is reported the total length (Mb) of ROHs per sample, and on the y-axis the coefficient of inbreeding (F). Note the different scale in each section. Bhutanese, Indian and Nepalese populations display the highest coefficient of inbreeding values and the highest total length of ROHs. Tibetan populations show the lowest coefficient of inbreeding and the lowest number of ROHs.

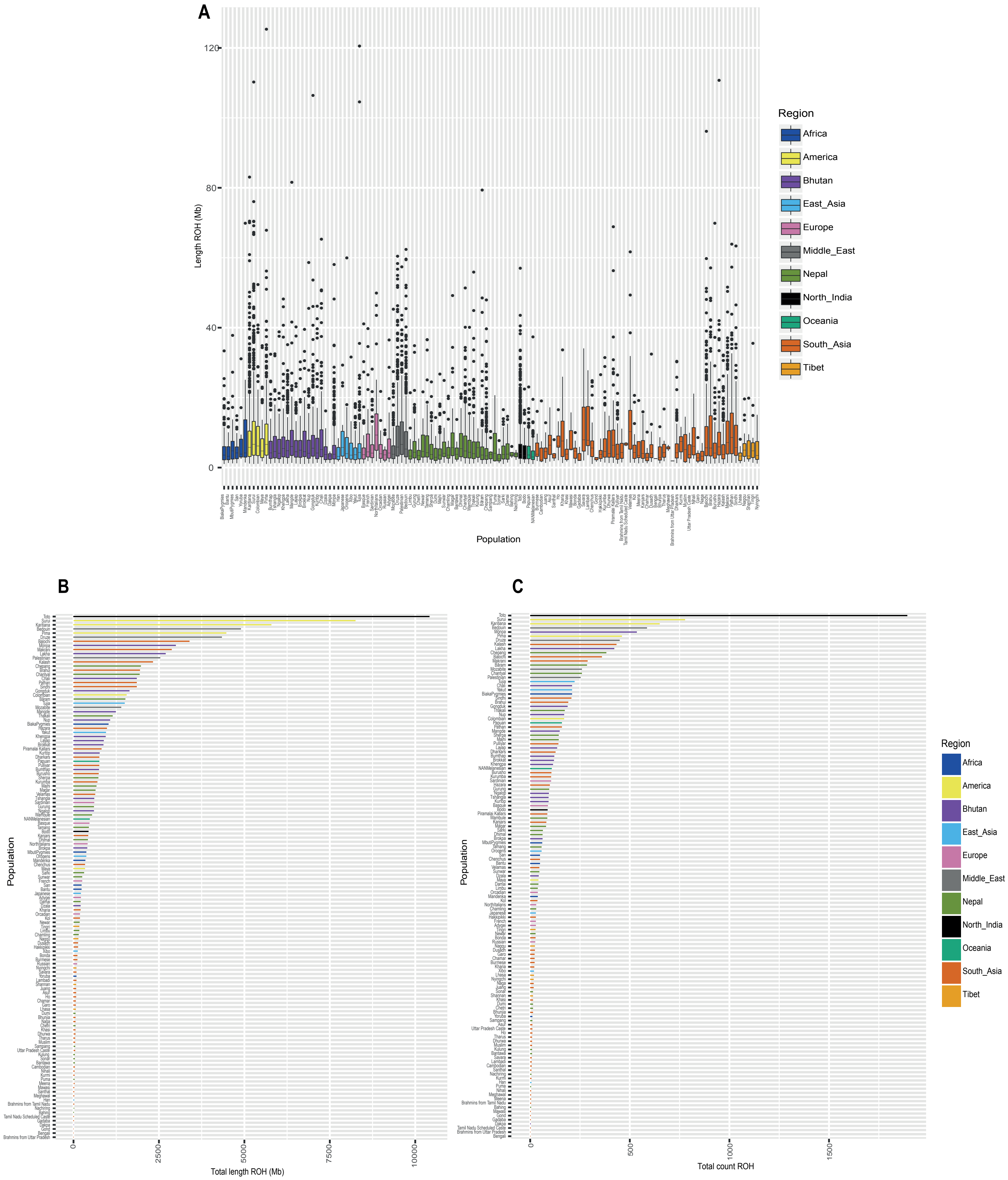

Fig. S9. Run of homozygosity (ROHs) in the worldwide populations. A. The x-axis reports the populations considered in the analysis and the y-axis represents the median length of ROHs in each population. The median length of ROHs in Himalayan populations is comparable with other worldwide populations. B, C. The x-axis displays total length of ROHs and total number of ROHs respectively, the y-axis represents the populations considered in the analysis. Toto shows the highest numbers of total length and number of ROHs.

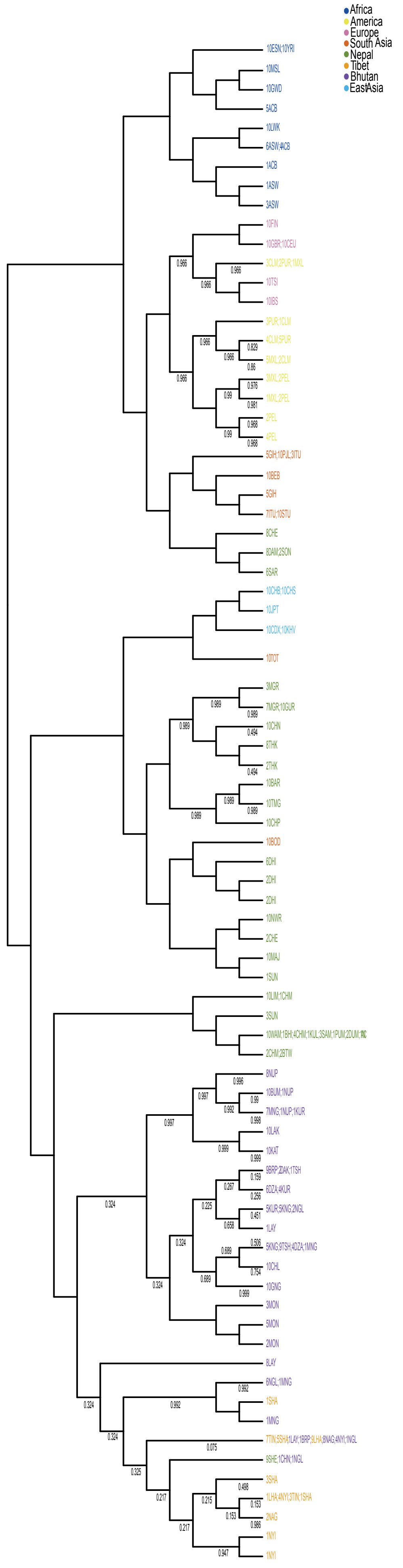

Fig. S10. fineSTRUCTURE phylogenetic tree of the Himalayan and the 1000 Genomes Project populations. Geographical clustering is noticeable. High altitude populations from Bhutan, Tibet and the Nepalese Sherpa cluster together.

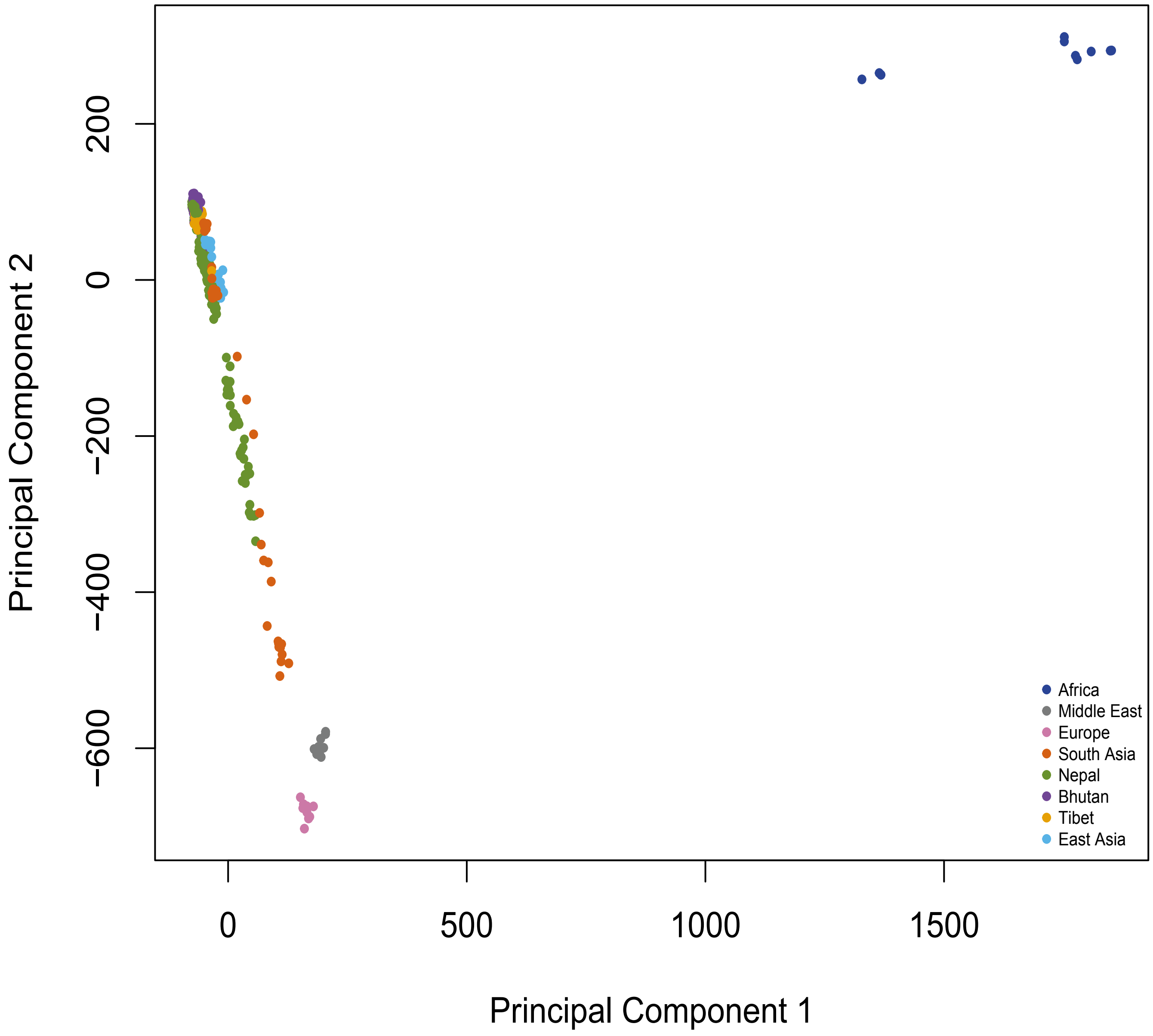

Fig. S11. PCA of fineSTRUCTURE co-ancestry matrix. The plot displays the Himalayan populations in the worldwide population context. Nepalese samples lie along a cline close to South Asians whereas Bhutanese and Tibetans cluster with East Asian populations.

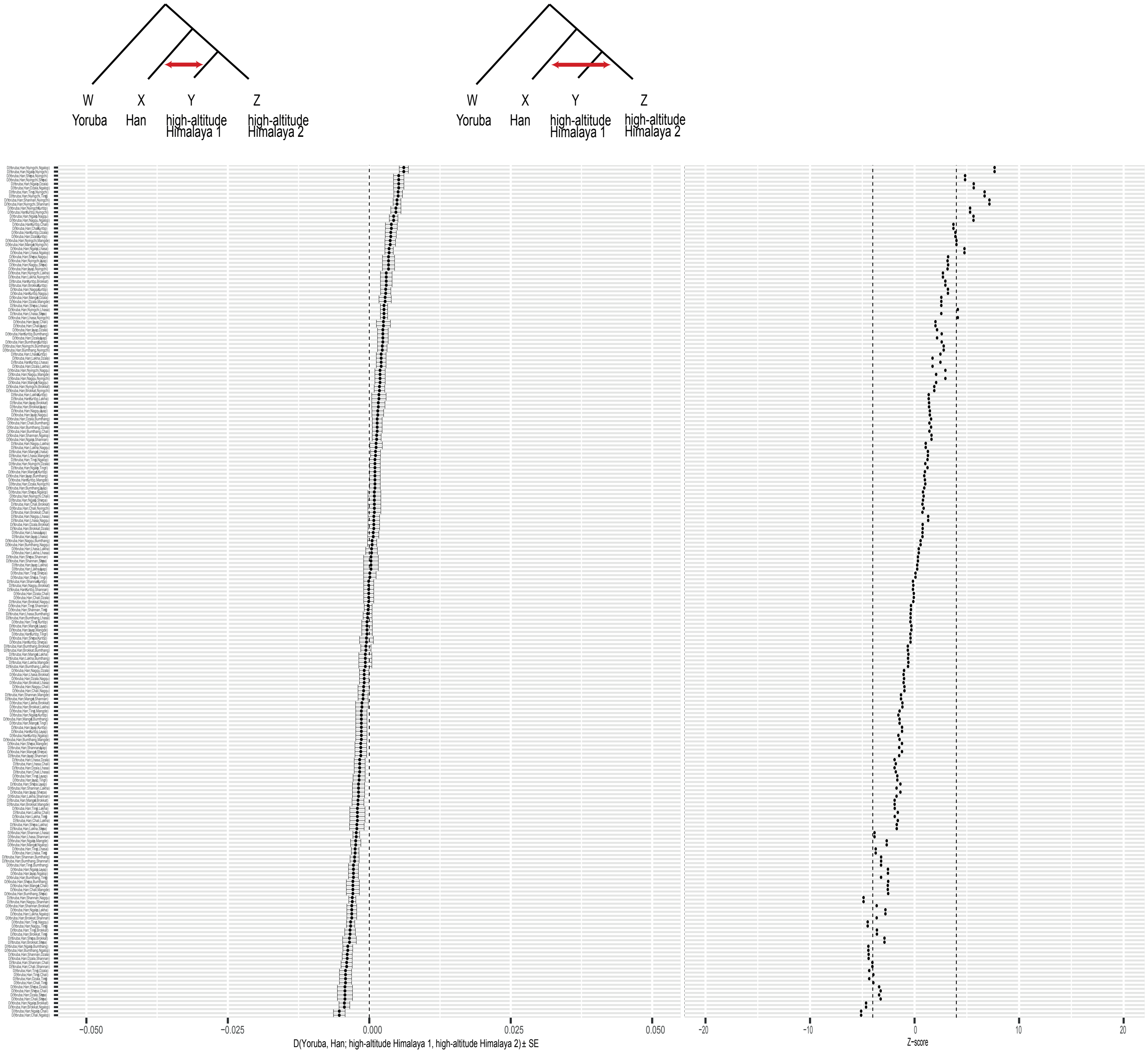

Fig. S12. Statistical analysis of genetic sharing between pairs of modern high-altitude Himalayan populations and Han individuals. The plot shows the results for the D-statistic test (x-axis) in the form *D*(Yoruba, Han; high-altitude Himalaya1, high-altitude Himalaya Himalaya2). Left: value of the *D*-statistic; right: associated *Z*-score for statistical significance. Values of *D*-statistics departing from zero and with *Z*-score in the extreme tail of the distribution (vertical dashed line in the plot) are indicative of more genetic sharing of one of the Himalayan populations with Han.

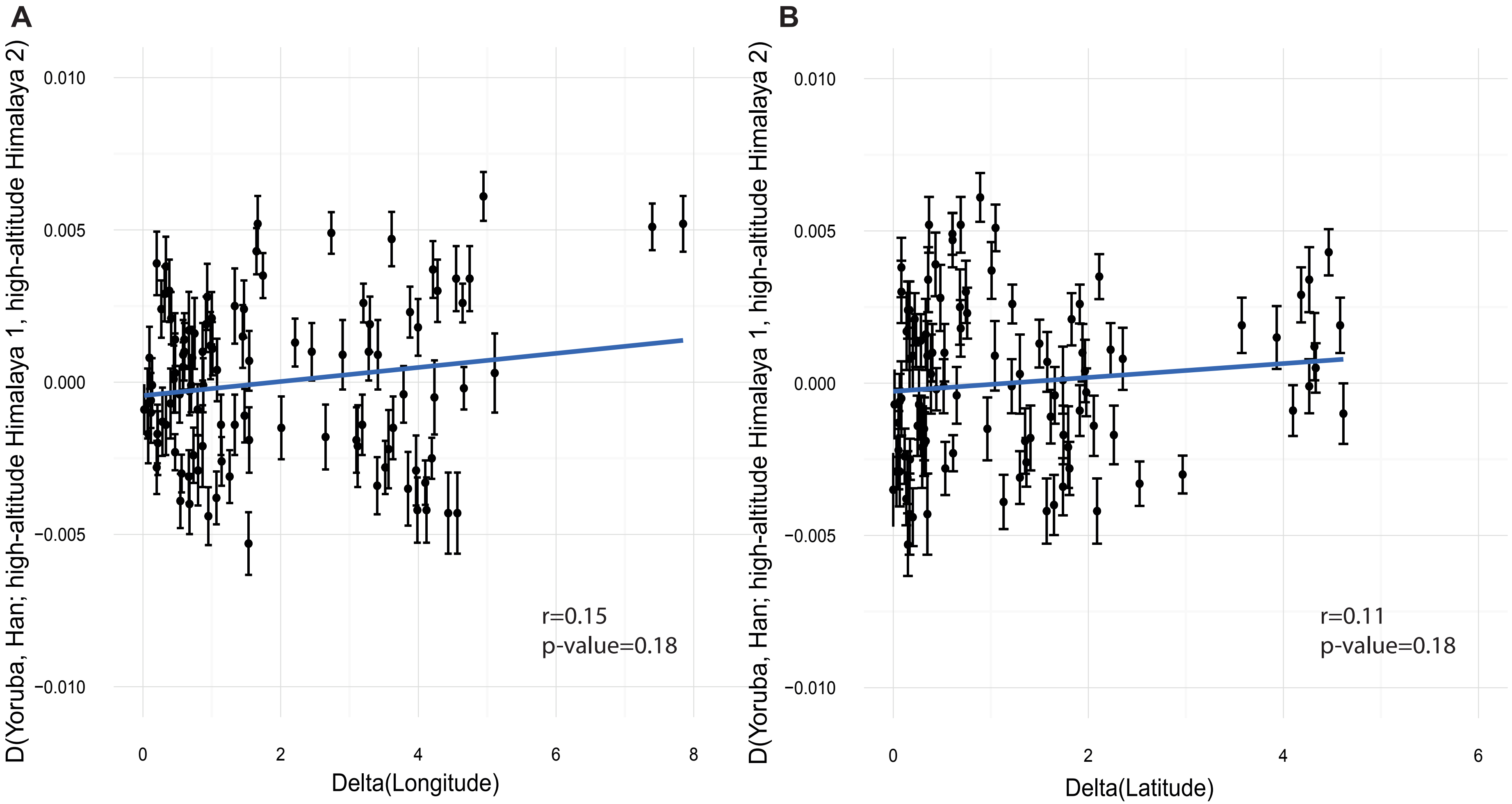

Fig. S13. Correlation plot of genetic and spatial distance between pairs of modern high-altitude Himalayan populations. The plot shows the results for D statistic test in the form of *D*(Yoruba, Han; high-altitude Himalaya1, high-altitude Himalaya2) (y-axis) compared with the difference in longitude (A) or latitude (B) for pairs of Himalayan populations (x-axis). The correlation coefficient (r) and the p-value are the results of the Mantel test between genetic distance (*D*-statistics values) and spatial distance (longitude and latitude).

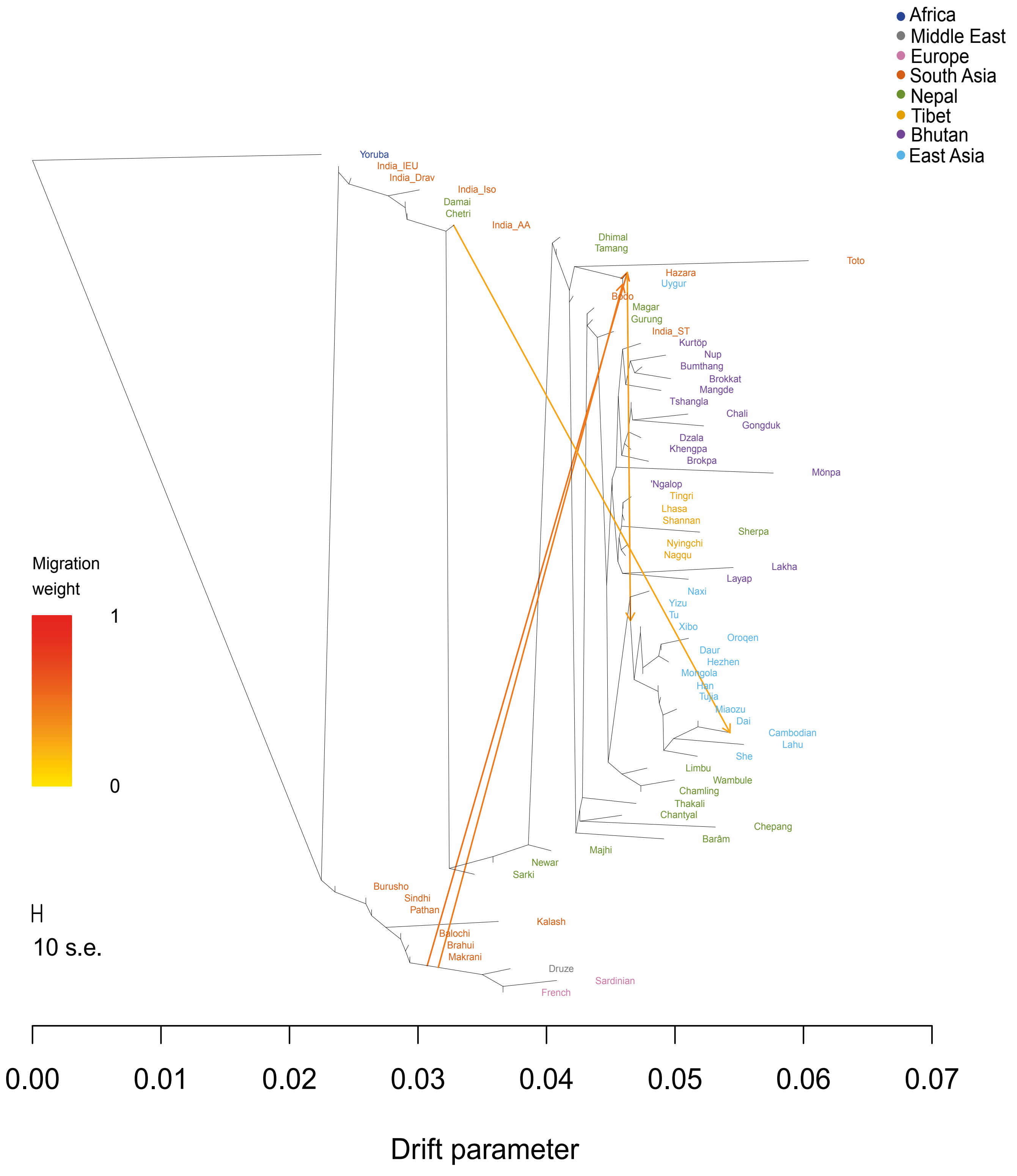

Fig. S14. TreeMix results from the worldwide dataset. The tree displays phylogenetic relationships and migration edges between the Himalayan populations and other worldwide populations. The x-axis represents the amount of genetic drift and migration edges are represented by arrows. The tree shows long branches for Toto, Mönpa and Chepang in agreement with their strong genetic drift patterns. Migration edges involving populations from South Asia, East Asia and Nepal are detectable. Dhimal and Bodo are characterised by migration events from South Asia (India and Pakistan).

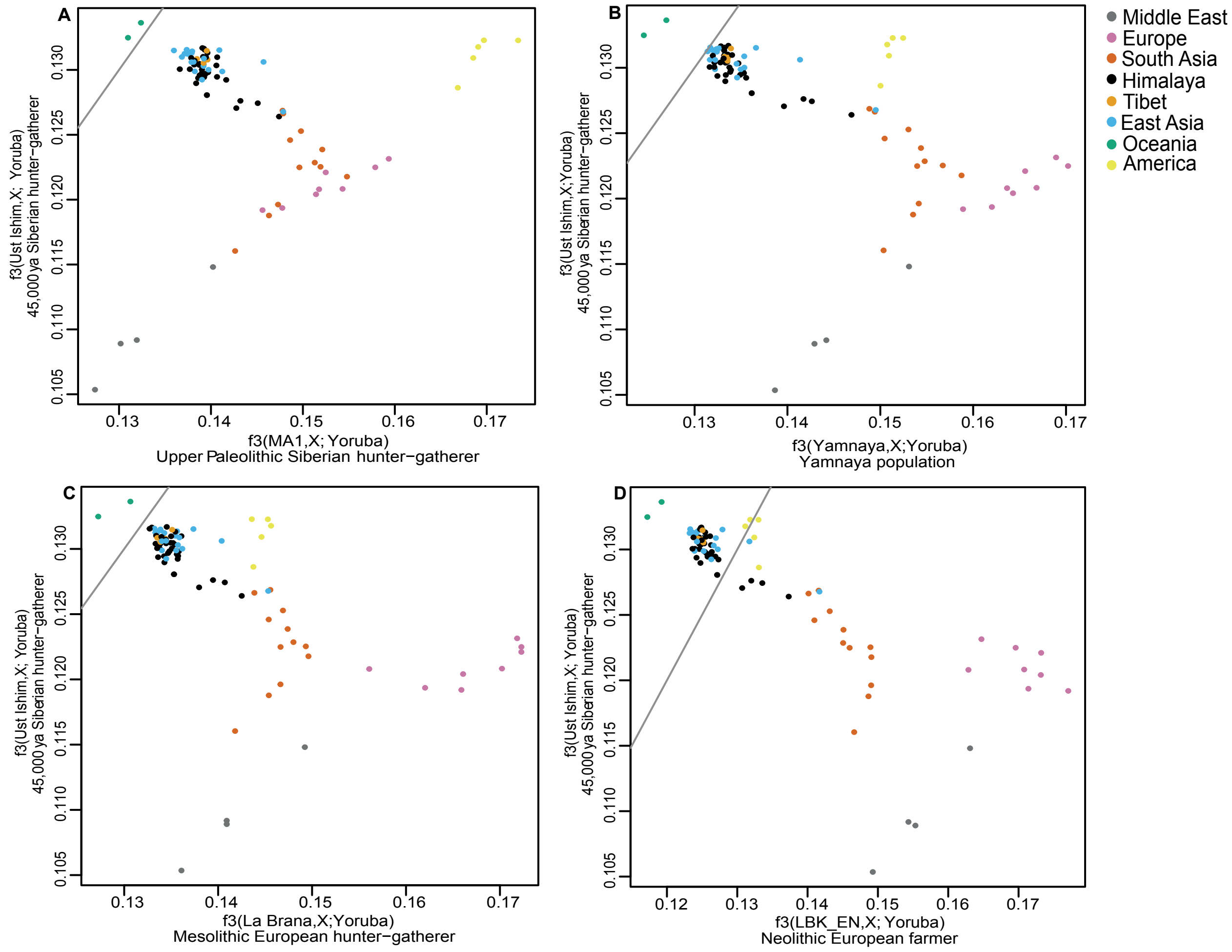

Fig. S15. Shared genetic drift within Himalayan populations and the 45,000-year-old Upper Paleolithic Siberian hunter-gatherer (Ust'-Ishim). In all the comparisons, Himalayan samples cluster with East and South Asians and have an equal genetic affinity with Ust'-Ishim and the other ancient genomes: 24,000-year-old Upper Paleolithic Siberian hunter-gatherer (MA-1), Yamnaya population, Mesolithic hunter-gatherer (La Braña) and Neolithic European farmers (LBK_EN).

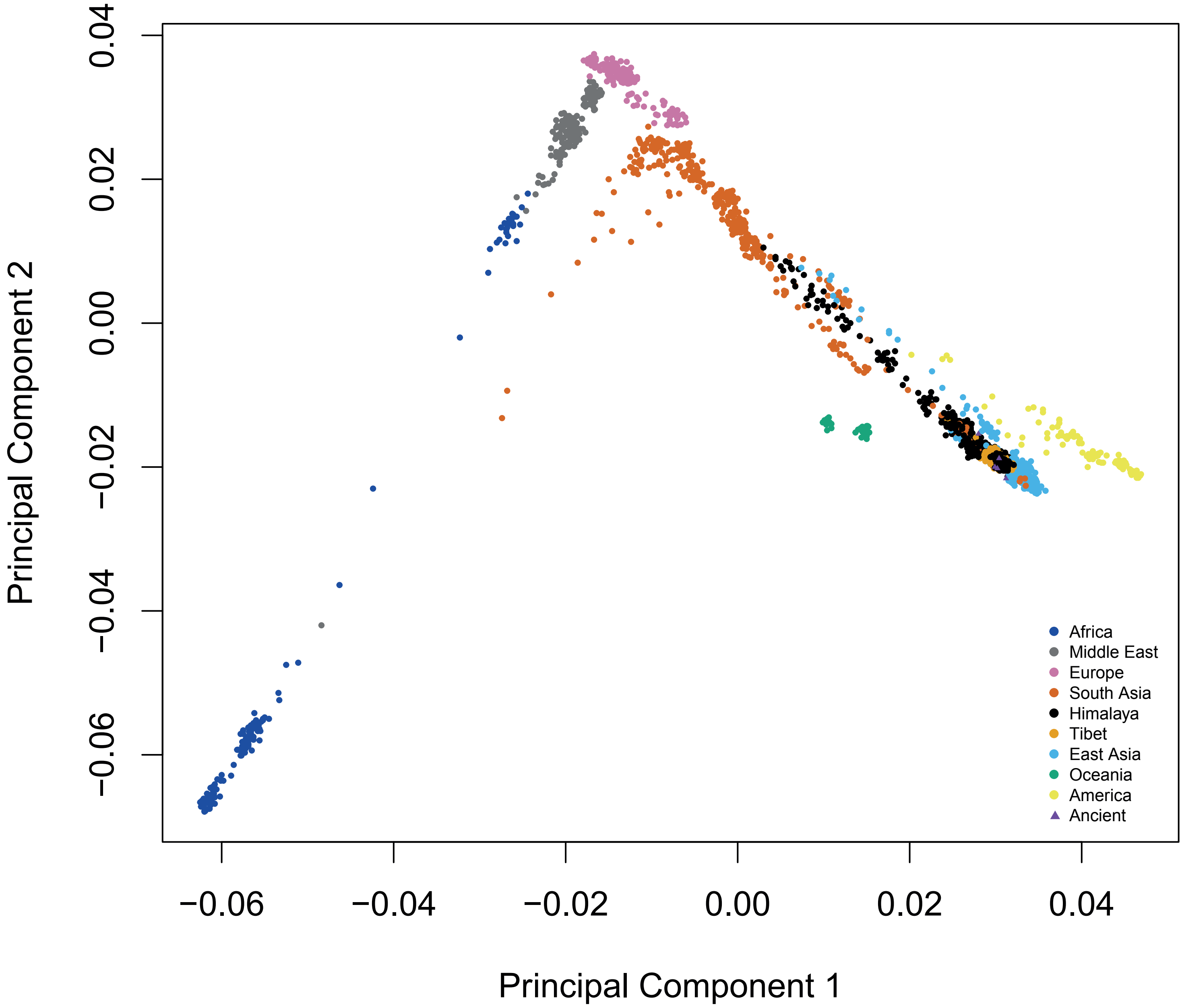

Fig. S16. PCA of the world dataset, with ancient Himalayans projected onto the plot. Ancient Himalayan samples (purple) cluster together with modern Himalayan populations.

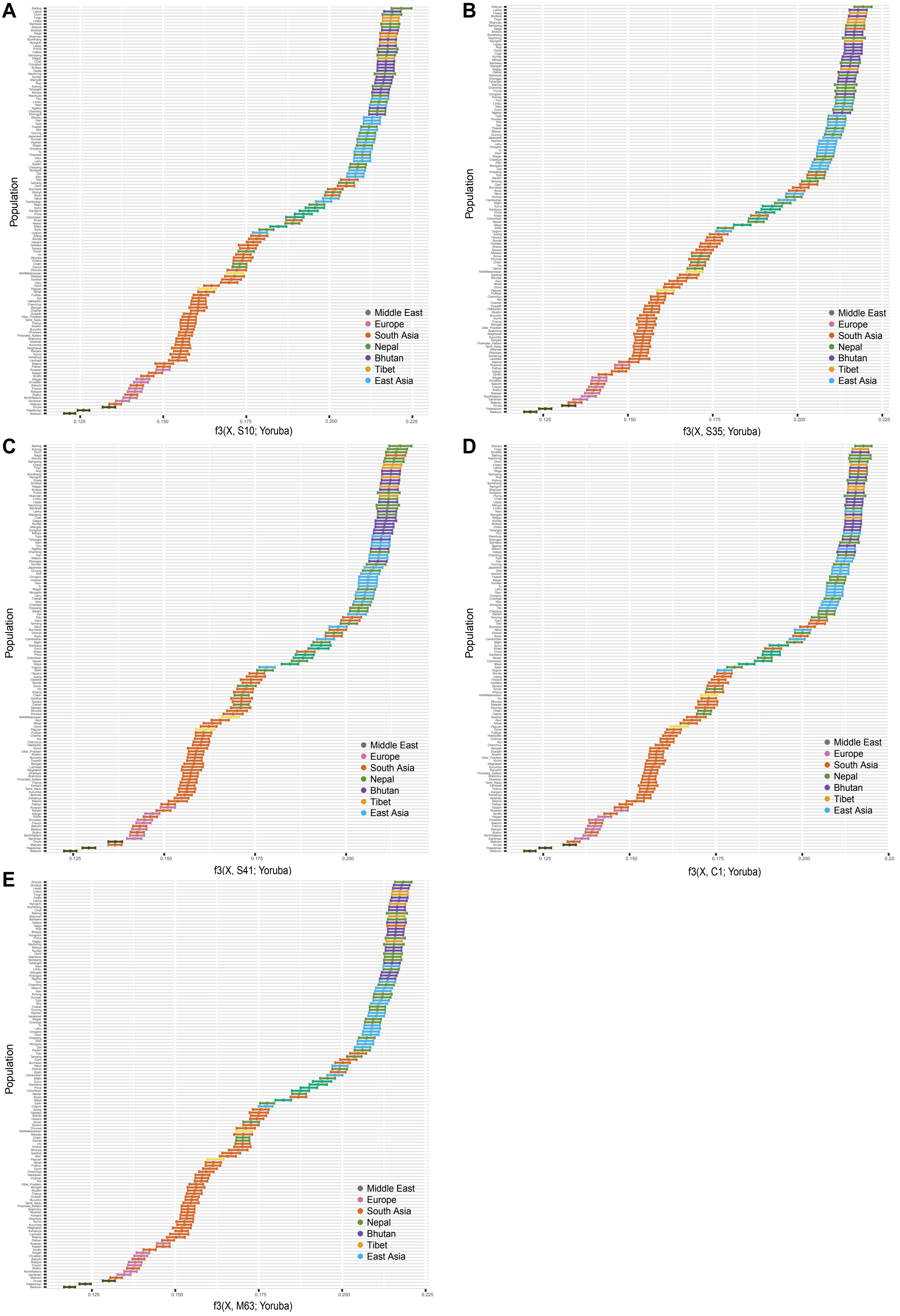

Fig. S17. Outgroup f3-statistics of the five ancient Himalayan individuals. A-E. The plots show shared genetic drift between modern populations and the ancient Himalayan individuals, S10, S35, S41, C1, M63. The x-axis represents the *f3*-statistic values and the y-axis reports the populations considered in the analysis.

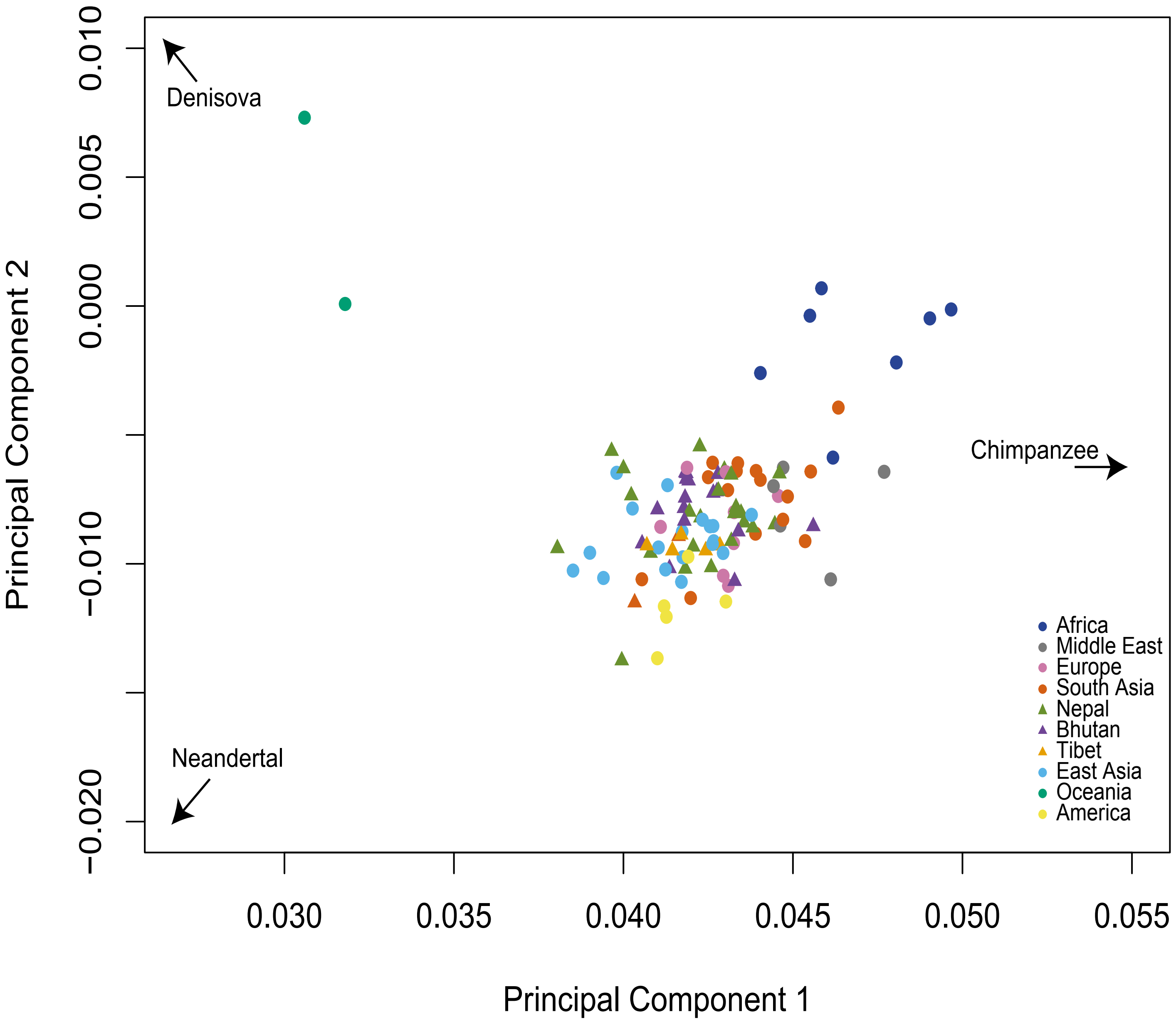

Fig. S18. PCA of modern human populations, Denisova and Neanderthal. The plot shows the projection of modern human samples onto principal components calculated using Denisova, Neanderthal and Chimpanzee. Himalayan populations show a similar pattern of genetic sharing with Denisova to the other South-East Asian populations.

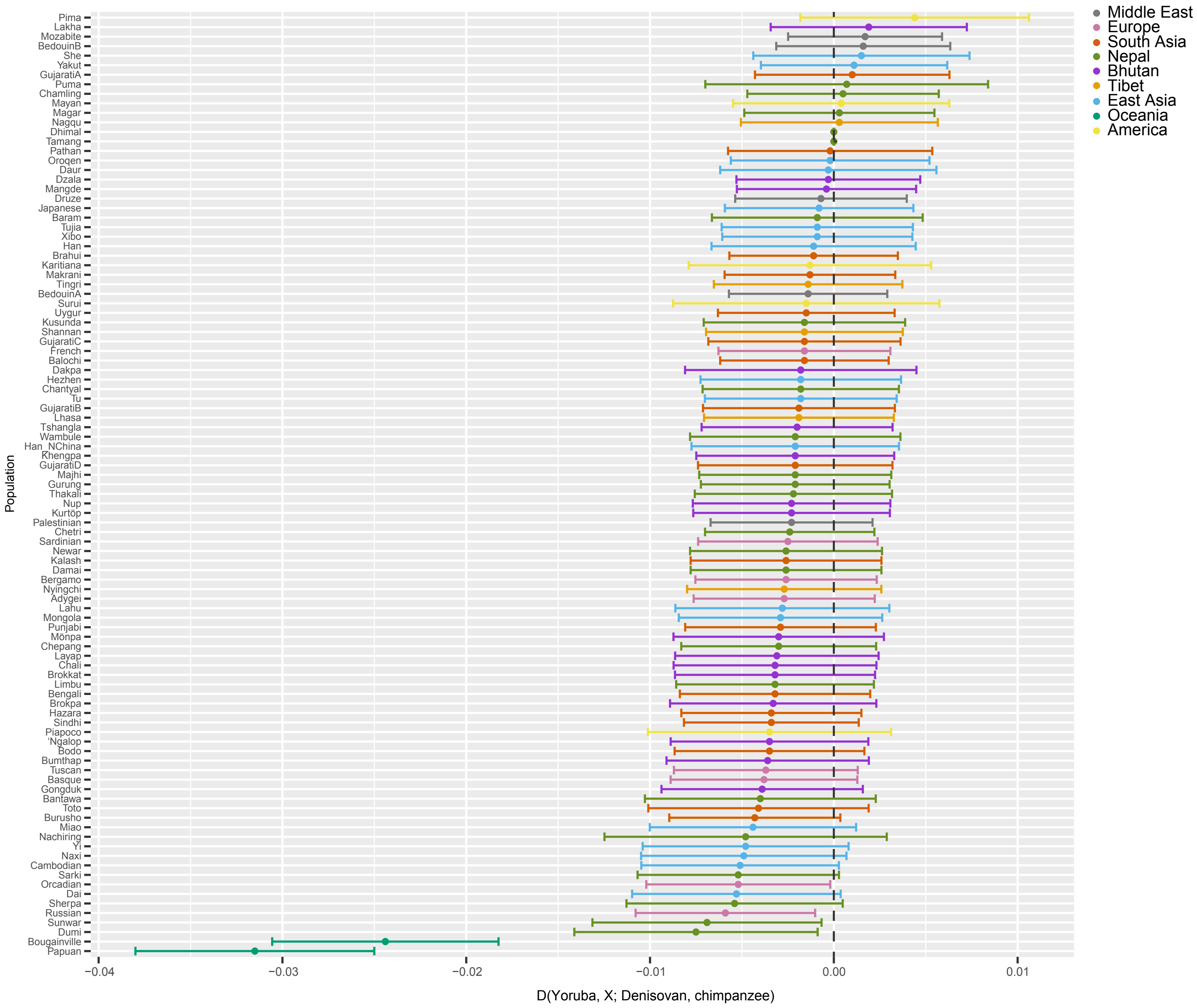

Fig. S19. Statistical analysis of genetic sharing between a modern human population and Denisova. The plot shows the results for the D-statistic test (x-axis) in the form *D*(Yoruba, X; Denisova, Chimpanzee) where X is any modern population considered in the analysis (y-axis).

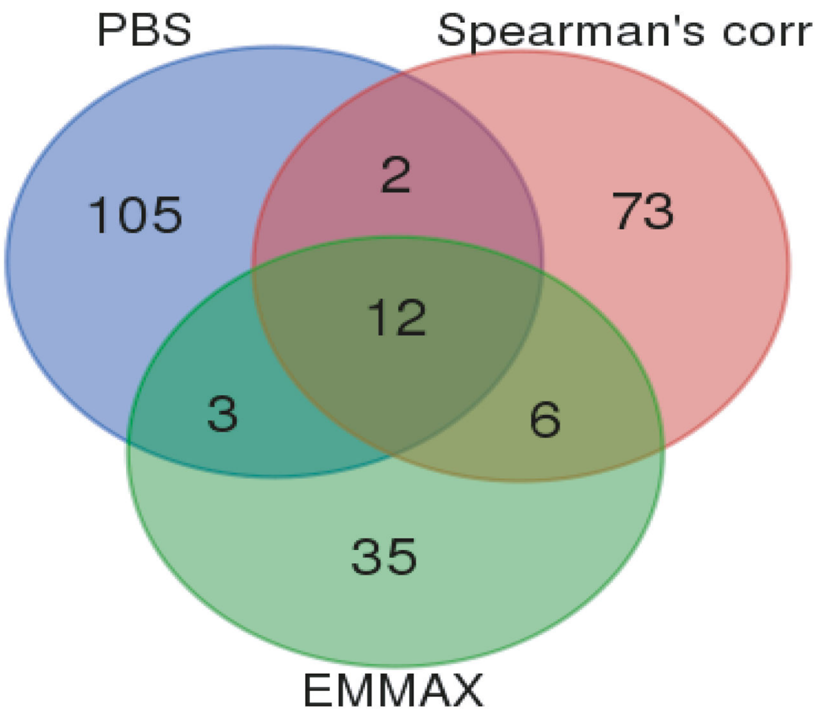

Fig. S20. Positive selection candidate overlaps between the three statistics used. Venn diagram of the overlaps between Spearman's correlation coefficient, EMMAX and PBS top candidates. Twelve candidate SNPs are shared by all three approaches.

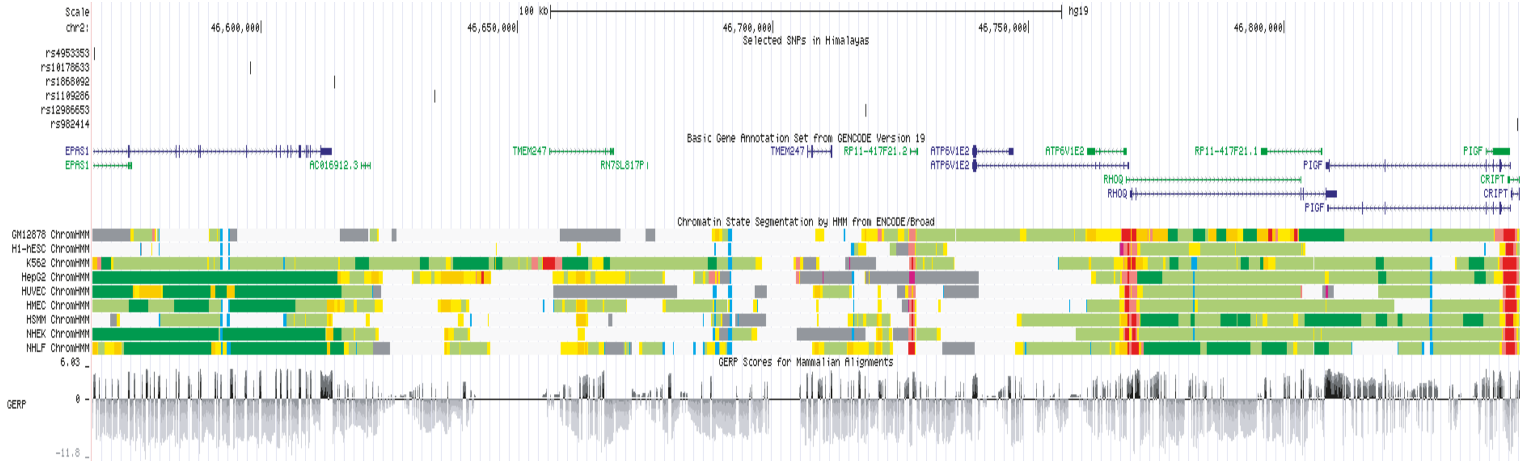

Fig. S21. Genomic features of a 278 kb region containing six candidate selected SNPs on chromosome 2. The plot shows the selection candidates in a linkage disequilibrium block (LD) with EPAS1.

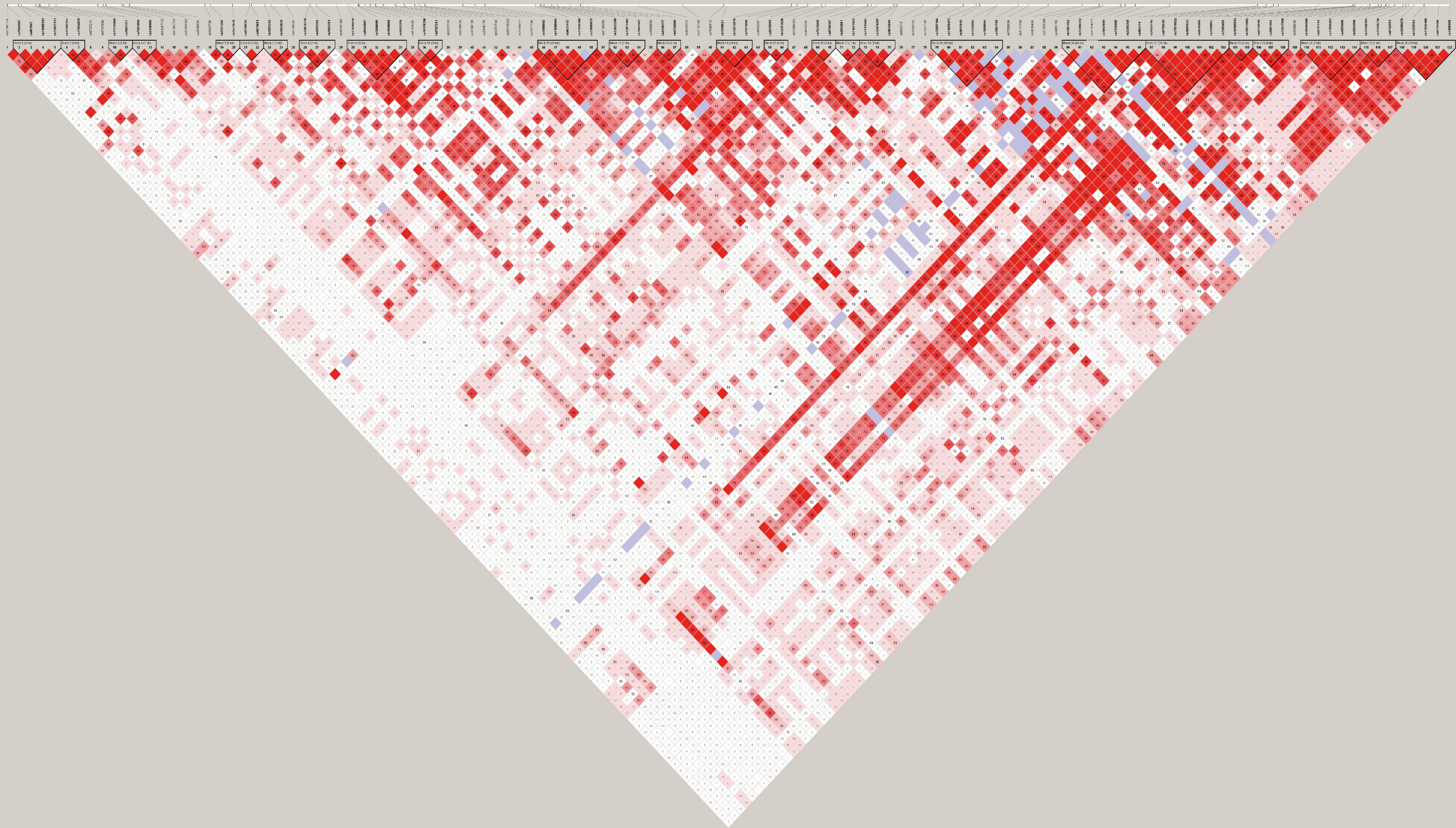

Fig. S22. LD plot of the region surrounding *EPAS1*. The plot shows the extent of linkage disequilibrium in a region spanning ~400kb surrounding *EPAS1*.

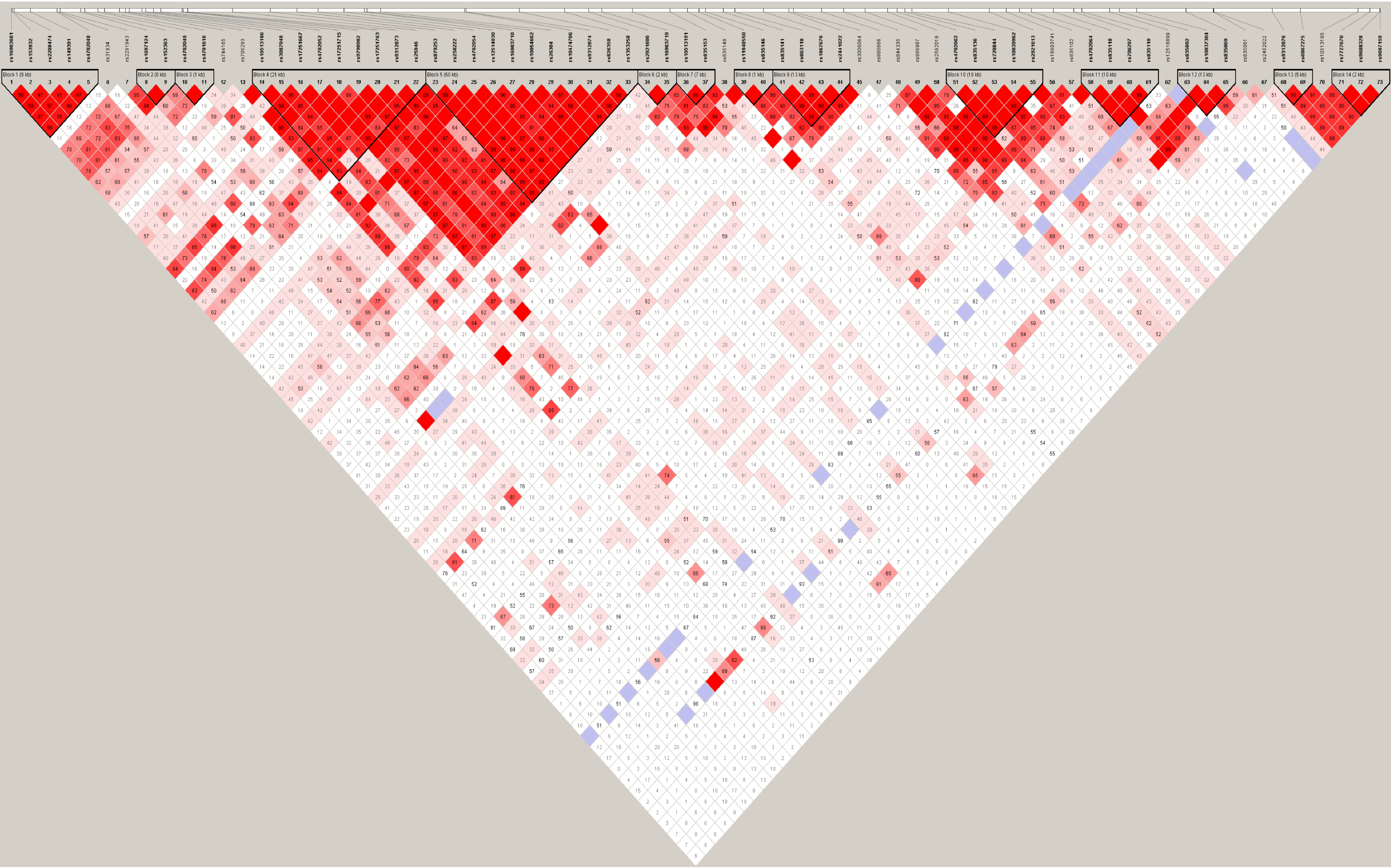

Fig. S23. LD plot of the region surrounding *ANKH* locus. The plot shows the extent of linkage disequilibrium in a region spanning ~ 300kb comprising *ANKH* gene and its upstream region on chromosome 5.

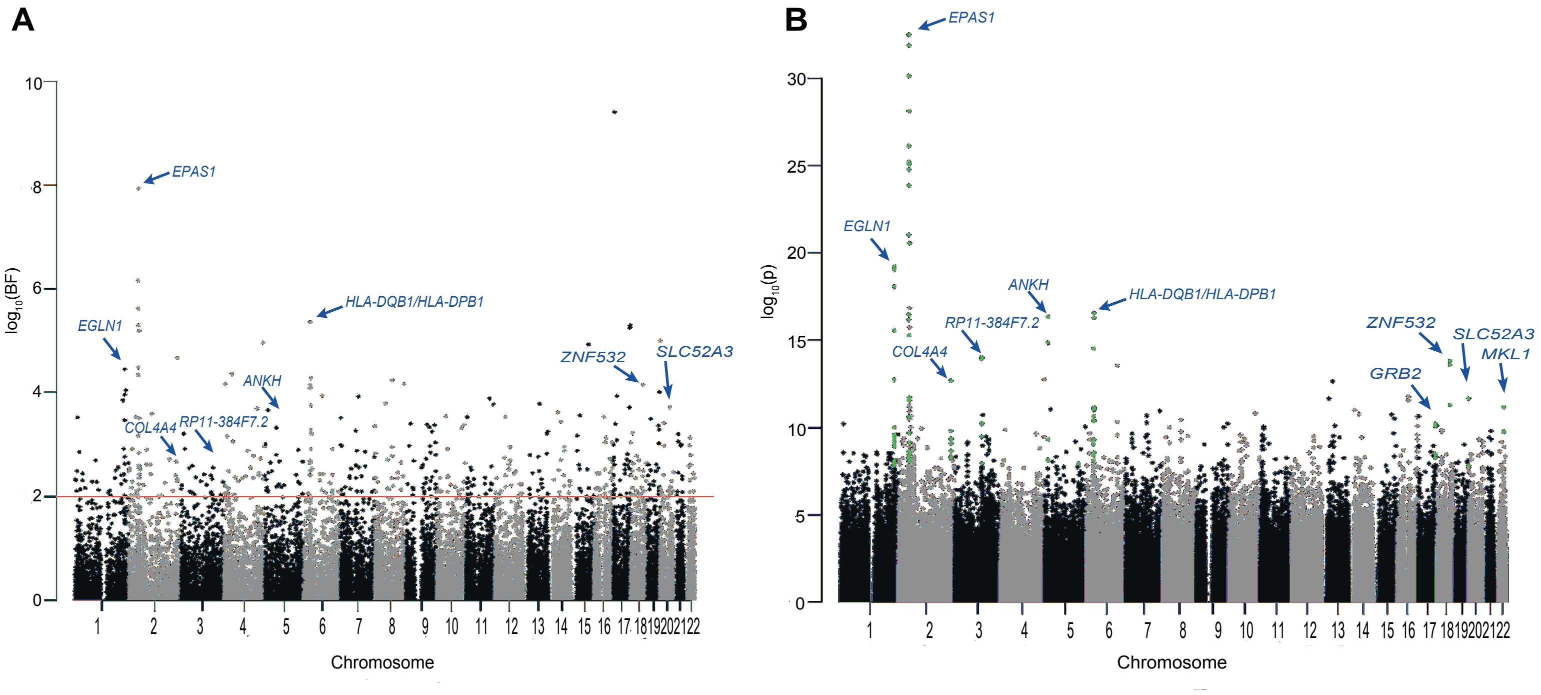

Fig. S24. Genome-wide signals of high-altitude adaptation in Himalayan populations. A, B. Manhattan plots displaying a measure of strength of selection signal (y- axis) plotted against genomic coordinate (x-axis). Each dot represents a SNP. A. BayEnv v2 results for the correlation between allele frequency and altitude. B. Fisher combined p-value for Spearman's correlation, EMMAX and PBS statistics. Eight out of ten candidate regions identified by Fisher's combined p value test are replicated by BayEnv v2.

SLC52A3

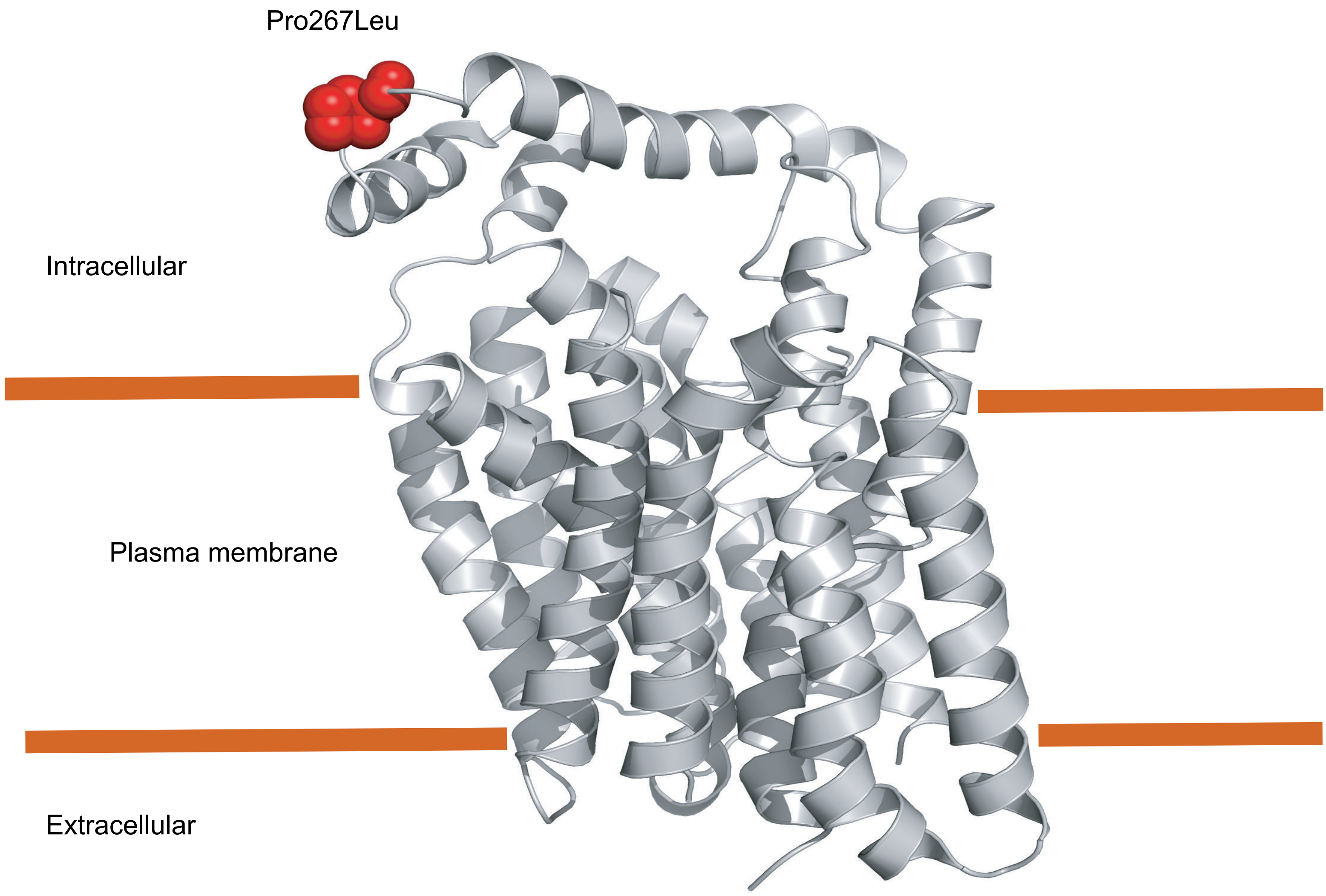

Fig. S25. SLC52A3 protein homology model. The plot shows the homology model generated for the SLC52A3 protein and its cellular transmembrane location. The missense variant, rs3746804, is indicated in red (Pro267Leu).

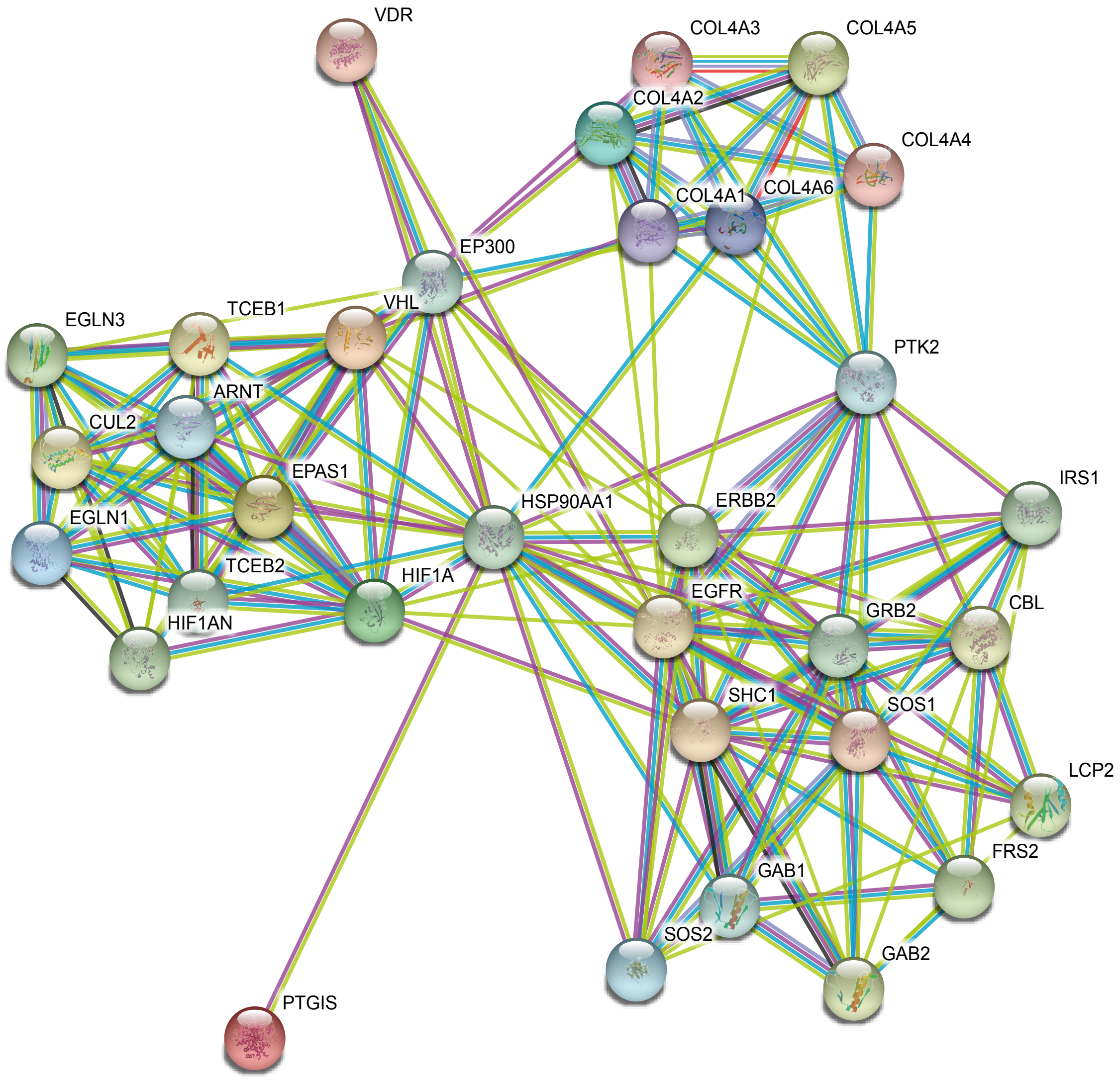

Fig. S26. Protein-protein interaction network of the top10 high-altitude adaptation candidates. *EPAS1*, *EGLN1*, *COL4A4* and *GRB2* are part of the same network together with two candidates, *PTGIS* and *VDR*, suggested by Hu et al. to also be under selection for high-altitude adaptation.
